# Fusarins G–L with Inhibition of NO in RAW264.7 from Marine-Derived Fungus *Fusarium solani* 7227

**DOI:** 10.3390/md19060305

**Published:** 2021-05-25

**Authors:** Guangyuan Luo, Li Zheng, Qilin Wu, Senhua Chen, Jing Li, Lan Liu

**Affiliations:** 1 School of Marine Sciences, Sun Yat-Sen University, Guangzhou 510006, China; luogy5@mail2.sysu.edu.cn (G.L.); zhengli26@mail2.sysu.edu.cn (L.Z.); wuqlin3@mail2.sysu.edu.cn (Q.W.); chensenh@mail.sysu.edu.cn (S.C.); cesllan@mail.sysu.edu.cn (L.L.); 2 Guangdong Provincial Key Laboratory of Marine Resources and Coastal Engineering, Zhuhai 519082, China; 3 Southern Marine Science and Engineering Guangdong Laboratory (Zhuhai), Zhuhai 519000, China; 4 Pearl River Estuary Marine Ecosystem Research Station, Ministry of Education, Zhuhai 519082, China

**Keywords:** *Fusarium solani*, fusarins, marine-derived fungus, anti-inflammatory activity

## Abstract

Six new fusarin derivatives, fusarins G–L (**1**–**6**), together with five known compounds (**5**–**11**) were isolated from the marine-derived fungus *Fusarium solani* 7227. The structures of the new compounds were elucidated by means of comprehensive spectroscopic methods (1D and 2D NMR, HRESIMS, ECD, and ORC) and X-ray crystallography. Compounds **5**–**11** exhibited potent anti-inflammatory activity by inhibiting the production of NO in RAW264.7 cells activated by lipopolysaccharide, with IC_50_ values ranging from 3.6 to 32.2 μM. The structure–activity relationships of the fusarins are discussed herein.

## 1. Introduction

**Figure 1:**
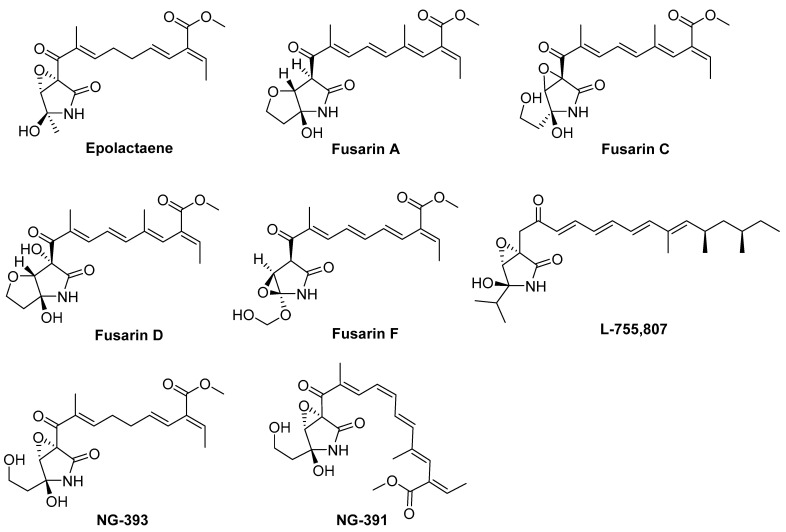
Structures of fusarin analogs.

*Fusarium* species are widely distributed in plants and soils worldwide. They have been proven to be an important source of plant pathogens in agriculture [1,2] and are also excellent sources of bioactive natural products. Among these, fusarin-related compounds are representative of *Fusarium* natural products, including epolactaene [3]; fusarins A, C, D, and F [4]; L-755,807 [5]; and NG-393 and NG-391 [6] (Figure 1), which possess a γ-lactam nucleus with a polyunsaturated side chain. *Fusarium* species have also received widespread attention in terms of both total chemical synthesis and biosynthesis [7,8]. For example, lucilactaene has been considered as a cell cycle inhibitor in p53-transfected cancer cells, which could arrest cell-cycle progression in a p53-independent manner in cells possessing a temperature-sensitive p53 protein, isolated from a strain of *Fusarium* species [9]. L-755,807 was reported as a bradykinin binding inhibitor, displaying bradykinin B2 receptor antagonist activity with an IC_50_ of 71 μM [5]. NG-391 has displayed the impairment of nucleic acid formation, while translation remains unaffected, in K-562 human cancer cells [10].

**Figure 2:**
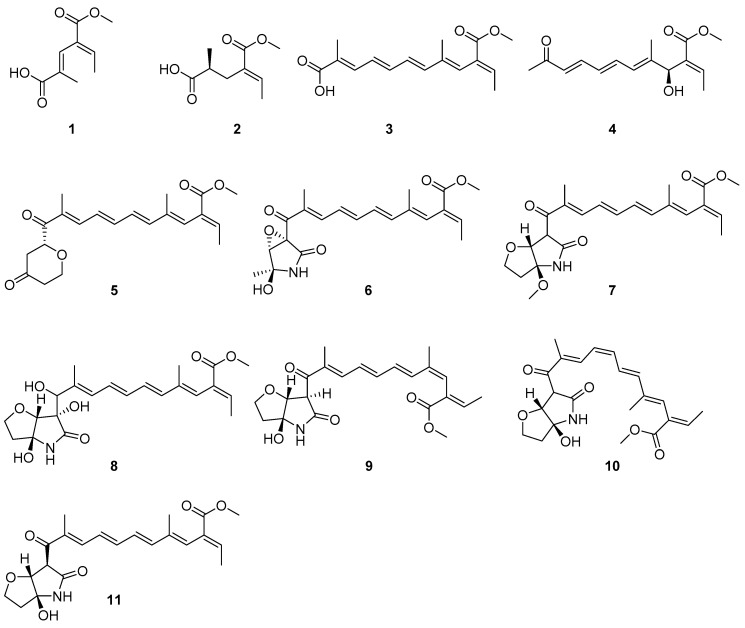
Structures of compounds **1**–**11.**.

To date, thirteen fusarin-related compounds (belonging to 3-oligoenoyltetramic acids) with a 1-oxopentadienyl substituent at C-3 in the tetramate ring have been discovered in marine microorganisms [11]. In fact, fusarin derivatives have rarely been reported in marine-derived fungi. Recently, the examination of extracts of *Fusarium solani* 7227, a seawater-derived fungus obtained from the South China Sea, led to the discovery of fusarin-related compounds. Further chemical investigation of this extract led to the isolation of six new fusarin derivatives (**1**–**6**) with a polyunsaturated side chain. According to the detailed spectroscopic analysis and by comparison with the reported literature data, the known compounds were identified as 3,5,7,9-undecatetraenoate (**7**) [12], methyl (2*E*,3*E*,5*E*,7*E*,9*E*)-11-((3*aS*,6*S*,6*aR*)-3*a*,6-dihydroxy-5-oxohexahydro-2*H*-furo[3,2-b]pyrrol-6-yl)-2-ethylidene-11-hydroxy-4,10-dimethylundeca-3,5,7,9-tetraenoate (**8**) [13], 4*Z*-lucilactaene (**9**), 8*Z*-lucilactaene (**10**), and lucilactaene (**11**) [14], as shown in Figure 2. Herein, we report the isolation, bioactive evaluation, and structure–activity relationships of compounds **1**–**11** from the marine-derived fungus *Fusarium solani* 7227.

## 2. Results

**Figure 3:**
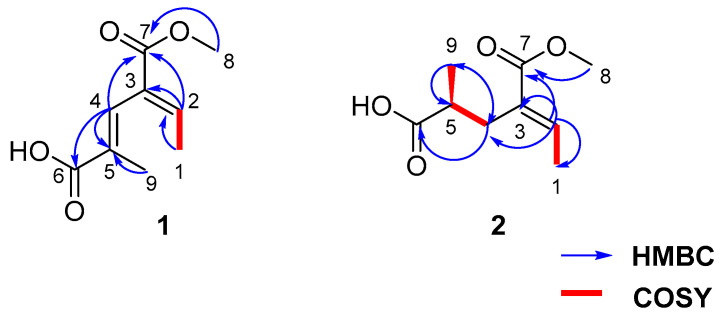
Structures of **1** and **2** with key COSY and HMBC correlations.

**Figure 4:**
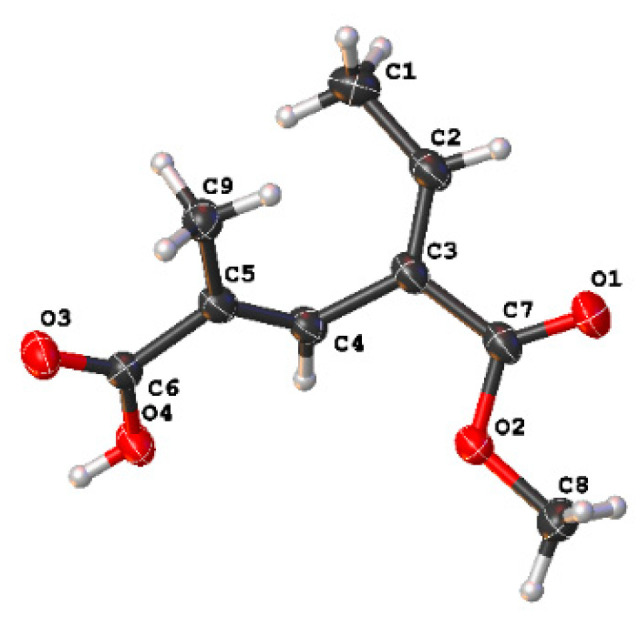
ORTEP representation of the X-ray crystal structure of **1**.

Fusarin G (**1**) was isolated as a white solid, and 6.4 mg was obtained from 197.9 g of crude extract. HRESIMS gave a molecular formula of C_9_H_11_O_4_, implying four degrees of unsaturation. Based on analysis of the ^1^H NMR data of **1** (Table 1), there were two olefinic proton signals, *δ*_H_ 7.08 (1H, qd, *J* = 7.2, 1.1 Hz) and 7.7 (1H, m); two methyls, *δ*_H_ 1.78 (3H, d, *J* = 1.4 Hz) and 1.76 (3H, d, *J* = 1.4 Hz); and one methoxy, H-8 *δ*_H_ 3.76 (3H, s). The planar structure of **1** was identified through ^1^H-^1^H COSY and HMBC analysis (Figure 3). The core moiety of compound **1**, a hexadienoic acid with a 5-substituted methyl, was established by means of HMBC correlations from H-4 to C-2, C-5, and C-6; H-9 to C-4, C-5, and C-6; and H-1 to C-2 and C-3; together with ^1^H-^1^H COSY data from H-1 and H-2. A methyl ester was linked to C-3, which was deduced from the HMBC correlation from H-8, H-4 and H-2 to C-7. The structure was further confirmed using X-ray crystallography. The X-ray structure (Figure 4) clearly showed that the geometric configurations were both *E*; two methyl groups on olefinic carbon were located on the same side but not on the same face with a small angle. The chemical shift of H-2 (*δ*_H_ 7.08) and H-4 (*δ*_H_ 7.36) were downfield shifts, due to the de-shielding effect of carbonyl C-7 (*δ*_C_ 166.7). This suggests that H-2 and H-4 are located on the same side as carbonyl C-7 in liquid.

Compound **2** was isolated as a white solid, and 7.3 mg was obtained from 197.9 g of crude extract. HRESIMS provided a molecular formula of C_9_H_14_O_4_. Analysis of the ^1^H NMR data (Table 1) indicated that the planar structure of **2** was similar to that of **1**. This was further supported by HMBC correlations (Figure S15) from H-2 to C-3, C-4, and C-7 and H-4 to C-5, C-6, and C-9, which also revealed the methyl C-9 to be connected to C-5. HMBC correlations also identified a methyl ester at C-3. Based on these data, the structure of **2** was determined as shown in Figure 2. To further identify the absolute configurations of compound **2**, we compared the calculated optical rotation of (5*S*)-**2** (+127.5) with the experimental data (+3.7), which indicated that the absolute configuration of **2** was (5*S*)-**2**. Compound **2** was named fusarin H.

Compound **3** was obtained as a yellow oil, and 9.8 mg was obtained from 197.9 g of crude extract. The molecular formula of C_16_H_20_O_4_ was deduced from its HRESIMS data. Compared with the NMR data (Table 2) of **2** and **3**, compound **3** shares a methyl butenoate fragment. The structural differences were apparent in the ^1^H-^1^H COSY NMR data (Figure S25) of **3**. These data revealed a spin system comprised of five olefinic hydrogens, H-6, H-7, H-8, H-9, and H-10, indicating that an unsaturated chain existed. HMBC (Figure S24) correlations from H-16 to C-10, C-11, and C-12 and from H-15 to C-6 and C-5 suggested that **3** had a dimethylundecatetraenoic acid chain linked to C-5. At the same time, HMBC correlations from H-4 to C-5 and C-13 were observed. This suggested that C-4 connected with the methyl butyrate moiety at C-3. Therefore, the structure of compound **3** was identified and named fusarin I.

**Figure 5:**
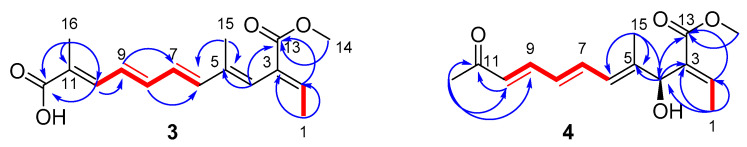
Structures of fusarins I (**3**) and J (**4**) with key COSY and HMBC correlations.

Compound **4** was isolated as a yellow amorphous solid, and 5.1 mg was obtained from 197.9 g of crude extract. The molecular formula was assigned as C_15_H_20_O_4_ based on HRESIMS data. Compared to the NMR data of **3** (Table 2), **4** showed similar spectroscopic features to **3**, which had an unsaturated chain and methyl butyrate moiety. The main difference in compound **4** was the absence of one olefinic proton and carboxyl acid, and the presence of an additional keto carbonyl and a secondary oxygenated carbon. HMBC correlations from H-12 to C-11, C-10, and C-9 revealed that keto carbonyl was located on C-11. In addition, a secondary alcohol was attached to C-4, determined on the basis of the chemical shift of C-4 (*δ*_C_ 71.8, *δ*_H_ 5.06) and the HMBC correlation of H-15 and H-1 with C-4. Hence, the planar structure of compound **4** was deduced, as shown in Figure 5. To further identify the absolute configurations of compound **4**, the calculated optical rotation of (4*S*)-**4** (+226.63) was compared with the experimental data (+25.2), which indicated the absolute configuration of **4** was *S*. Finally, compound **4** was named fusarin J.

**Figure 6:**
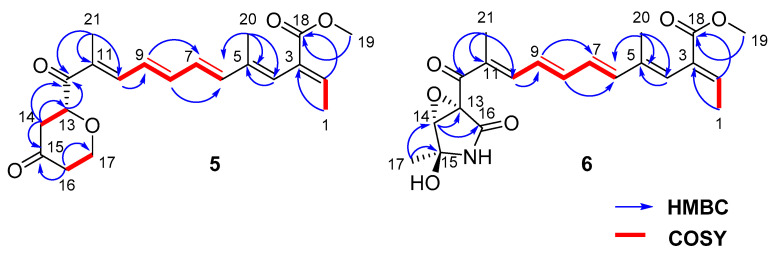
Structures of fusarins K (**5**) and L (**6**) with key COSY and HMBC correlations.

Compound **5** was obtained as a yellow amorphous substance, and 1.7 mg was obtained from 197.9 g of crude extract. The molecular formula was assigned as C_26_H_26_O_5_ based on HRESIMS data. The 1D NMR data (Table 3) indicated that compounds **5** and **3** had common fragments, except for the change in the carboxyl group C-12 (*δ_C_* 196.94), replaced with a *γ*-pranone unit, which was supported by the ^1^H-^1^H COSY (Figure 6) of H-13, H-14 and H-16, H-17, with two spin systems and HMBC correlations (Figure 6) from H-14 to C-12, C-13, and C-15; H-16 to C-15; and H-17 to C-13. To further identify the absolute configurations of compound **5,** the calculated optical rotation of (13*S*)-**5** (−309.39) was compared with the experimental data (+1.8), which indicated the absolute configuration of **5** was *R*. Compound **5** was named fusarin K.

**Figure 7:**
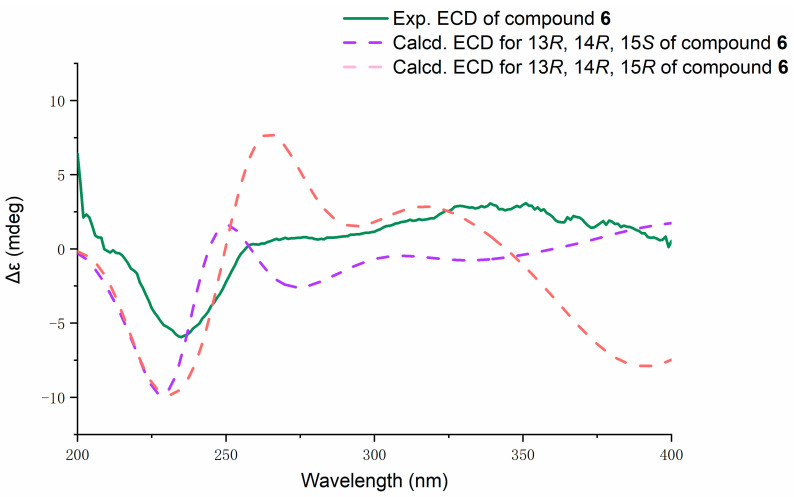
Experimental ECD spectrum of **6** (green) in methanol with computed spectra of the enantiomers 13*R*, 14*R*, and 15*R* and 13*R*, 14*R*, and 15*S* at the b3lyp/6-31+g(d,p) level of theory. Red dotted line: 13*R*, 14*R*, and 15*R*; purple dotted line: 13*R*, 14*R*, and 15*S*.

Fusarin L (**6**) was obtained as a yellow amorphous substance, and 11.3 mg was obtained from 197.9 g of crude extract. HRESIMS analysis gave a molecular formula of C_21_H_24_NO_6_ with 10 degrees of unsaturation. Based on the analysis of 1D and 2D NMR data (Table 3), compound **6** belongs to a fusarin derivative and has a similar structure to that of **5** in the comparison of their NMR data. The HMBC correlations from methyl proton H-17 to C-15 and C-14 and from H-14 to C-13 and C-16 indicated an epoxy-*γ*-lactam ring with a methyl at C-15, which was linked to keto carbonyl C-11 in compound **6,** which replaced a pyrone moiety in compound **5**. Hence, the planar structure of **6** was confirmed. The theoretical ECD spectra (Figure 7) of compound **6** were calculated by means of a quantum chemical method at the [b3lyp/6-31+g(d,p)] level for a pair of configurations—13*R*, 14*R*, and 15*S* and 13*R*, 14*R*, and 15*R*. The computed configurations of 13*R*, 14*R*, and 15*S* were well in agreement with the experimental results. Furthermore, the computed optical rotation of (13*R*, 14*R*, 15*S*)-**6** with +351.83 was consistent with the experimental value (+44.95). Thus, combined with ECD, ORC, and the literature [13], the absolute configuration of **6** was proposed, with stereochemical assignments of 13*R*, 14*R*, and 15*S*.

All the compounds were evaluated for their inhibition of nitric oxide (NO) production in RAW264.7 cells activated by lipopolysaccharide (LPS) using the Griess assay with indomethacin as a positive control (Table 4). Compounds **9**, **10**, and **11** displayed strong inhibition of nitric oxide (NO) production, with IC_50_ values of 7.6, 3.6, and 8.4 μM, respectively, whereas compounds **2**, **3**, **4**, and **5** showed moderate anti-inflammatory activity, with IC_50_ values of 22.3, 13.1, 13.9, and 21.9 μM. In comparing the anti-inflammatory activity of fusarins G and H, **2** showed much stronger activity than that of **1**, indicating that the double bond (C4–C5) of **1** is a disadvantage in terms of anti-inflammatory activity. Compound **5** exhibited stronger anti-inflammatory effects than those of **6**, indicating that the pyrone moiety made a more positive contribution to the anti-inflammatory activity than the epoxy-γ-lactam ring.

## 3. Materials and Methods 

### 3.1. General Experimental Procedures

One-dimensional and two-dimensional NMR spectra were acquired in CDCl_3_ with a Bruker Avance 400 MHz NMR spectrometer (Fällanden, Switzerland). HR–ESI–MS data were measured using a maXis Q-TOF mass spectrometer in positive ion mode (Bruker, Fällanden, Switzerland). UV and CD spectra were acquired using a JASCO J-810 circular dichroism spectrometer (JASCO International Co. Ltd., Hachioji, Tokyo, Japan). X-ray diffraction intensity data were recorded on an XtalLAB PRO single-crystal diffractometer, using Cu-Kα radiation (Rigaku, Japan). The data were corrected for absorption, using CrysAlisPro 1.171.39.33c (Rigaku Oxford Diffraction, 2017). Optical rotations were acquired using an MCP-500 polarimeter with a 1.0 mL cell (Anton, Austria). HPLC was performed on the Hitachi Primaide with the YMC ODS SERIES column (YMC-Pack ODS-A, YMC Co. Ltd., Kyoto, Japan, 250 × 10 mm I.D., S-5 μm, 12 nm). Column chromatography (CC) was carried out on silica gel (200–300 mesh, Qingdao Marine Chemical Factory, Qingdao, China) and Sephadex LH-20 (40–70 μm, Amersham Pharmacia Biotech AB, Uppsala, Sweden). TLC plates with silica gel GF254 (0.4–0.5 mm, Qingdao Marine Chemical Factory, Qingdao, China) were used for the analyses.

### 3.2. Fungal Isolation and Fermentation

*Fusarium solani* 7227 was isolated from a seawater sample collected in February 2018 from the South China Sea. The fungus was identified as *Fusarium solani* by morphological observation and analysis of the internal transcribed spacer (ITS) regions of its rDNA, the sequence data of which were deposited in GenBank with the accession number MN922526. The strain was grown on PDA plates at 28 °C for 5 days; then it was cut into small pieces and inoculated into 1.5 L of PDA medium as a seed liquid at 28 °C for 2 days in a shaker; then poured in 5–10 mL of seed liquid into 180 6-inch plates of sterilized rice medium (rice 60 g, 0.3% salinity water 60 mL) for fermentation at 28 °C for 28 days.

### 3.3. Extraction and Isolation

The fermented material was extracted with EtOAc under ultrasonication, then concentrated under reduced pressure to afford 197.9 g of a crude extract. The crude extract was subjected to silica gel column chromatography (CC) (9 × 40 cm, 700 g), using gradient mixtures of PE (petroleum ether)/EtOAc (60:1 to 1:9), followed by 100% CH_3_OH, to afford 4 major subfractions (Fr. 1–Fr. 4).

Fr. A was fractionated using CC on silica gel (300–400 mesh) and eluting with PE/EA (from 9:1 to 1:1), to afford 2 fractions, Fr. A-1 and Fr. A-2. Those two fractions were further separated on Sephadex LH-20 (CH_2_Cl_2_/MeOH *v*/*v*, 1:1). Fr. A-1 was purified by means of NP-HPLC (n-hexane: IPA (isopropyl alcohol) *v*/*v*, 99:1, flow rate 2.0 mL/min) to obtain **1** (t_R_ 24.5 min, 4.6 mg) and **2** (t_R_ 21.9 min, 7.3 mg). Fr. A-2 was purified by means of NP-HPLC (n-hexane: IPA *v*/*v*, 97:3, flow rate 2.0 mL/min) to obtain **3** (t_R_ 21.2 min, 9.8 mg). Fr. B was separated with Sephadex LH-20 (CH_2_Cl_2_/MeOH *v*/*v*, 1:1) to afford Fr. B-1 and Fr. B-2. Fr. B-1 was further purified by means of NP-HPLC (n-hexane: IPA *v*/*v*, 94:6, flow rate 2.0 mL/min) to obtain **4** (t_R_ 21.2 min, 5.1 mg), and Fr. B-2 was purified by means of NP-HPLC (n-hexane: IPA *v*/*v*, 9:1, flow rate 2.0 mL/min) to obtain **5** (t_R_ 19.1 min, 1.7 mg). Compounds **1**–**5** were purified by means of analytical NP-HPLC (Venusil XBP Silica column 4.6 × 250 mm, 5 μm). Fr. C was subjected to Sephadex LH-20 (CH_2_Cl_2_/MeOH *v*/*v*, 1:1) to obtain Fr. C-1 and Fr. C-2. Fr. C-1 was subjected to silica gel CC with PE/EA (from 4:1 to 2:3), resulting in two further subfractions, Fr. C-1-1 and Fr. C-1-2. Compound **6** (t_R_ 14.0 min, 11.3 mg) was separated by means of semipreparative NP-HPLC (Venusil XBP Silica column 10 × 250 mm, 5 μm) and eluted with n-hexane: IPA *v*/*v*, 94:6 with flow rate 2.0 mL/min from Fr. C-1-1. The purification of Fr.1-2 was performed by means of RP-HPLC (H_2_O: MeOH *v*/*v*, 3:7, flow rate, 4.0 mL/min), affording compounds **9** (t_R_ 10.2 min, 2.8 mg) and **10** (t_R_ 8.2 min, 5.3 mg). Fr. C-2 was subjected to the same process as that used for Fr. C-1, affording Fr. C-2-1 and Fr. C-2-2. Fr. C-2-1 was purified by means of RP-HPLC (H_2_O: MeOH *v*/*v*, 34:66, flow rate 4.0 mL/min), affording compounds **7** (t_R_ 13.8 min, 6.9 mg) and **11** (t_R_ 11.0 min, 6.1 mg). Fr. C-2-2 was purified by means of RP-HPLC (H_2_O: MeOH *v*/*v*, 4:1, flow rate 3.0 mL/min), affording compound **6** (t_R_ 9.5 min, 11.3 mg). Compounds **6**–**11** were purified by means of analytical NP-HPLC (Venusil XBP Silica column 4.6 × 250 mm, 5 μm).

Compound **1**: colorless crystal; UV (MeOH) λmax (log ε) 203 (4.2) nm; IR (neat) *ν*_max_ 723, 761, 817, 904, 943, 979, 1025, 1060, 1135, 1267, 1328, 1384, 1438, 1637, 1718, 2954 cm^−1^ (Figure S1); ^1^H NMR (CDCl_3_, 400 MHz) and ^13^C NMR (CDCl_3_, 100 MHz), see Table 1; HRESIMS *m*/*z* 183.0662 [M − H]^−^ (calcd. for C_9_H_11_O_4_, 183.0663).

Compound **2**: Yellow amorphous solid; [α${]}_{D}^{25}$ +3.7 (c 0.1, MeOH); UV (MeOH) *λ*max (log ε) 212 (3.6) nm; IR (neat) *ν*_max_ 767, 825, 860, 892, 952, 993, 1026, 1093, 1213, 1249, 1272, 1378, 1405, 1438, 1459, 1712, 2854, 2925, 3438 cm^−1^ (Figure S18); ^1^H NMR (CDCl_3_, 400 MHz) and ^13^C NMR (CDCl_3_, 100 MHz), see Table 1; HRESIMS *m*/*z* 185.08198 [M − H]^−^ (calcd. for C_9_H_13_O_4_, 185.08193).

Compound **3**: Yellow amorphous solid; UV (MeOH) λmax (log ε) 335(3.8), 212(3.5) nm; IR (neat) *ν*_max_ 1413, 1467, 1629, 1652, 2844, 2914, 3180, 3349 cm^−1^ (Figure S27); ^1^H NMR (CDCl_3_, 400 MHz) and ^13^C NMR (CDCl_3_, 100 MHz), see Table 2; HRESIMS *m*/*z* 275.12920 [M − H]^−^ (calcd. for C_16_H_19_O_4_, 276.12888).

Compound **4**: Yellow amorphous solid; [α${]}_{D}^{25}$ +25.2 (c 0.1, MeOH); UV (MeOH) λmax (log ε) 208(3.5), 270(3.2), 323(3.3) nm; IR (neat) *ν*_max_ 763, 902, 964, 998, 1045, 1072, 1133, 1191, 1255, 1294, 1361, 1436, 1515, 1573, 1602, 1637, 1664, 1708, 2852, 2917, 2952, 3457 cm^−1^ (Figure S36); ^1^H NMR (CDCl_3_, 400 MHz) and ^13^C NMR (CDCl_3_, 100 MHz), see Table 2; HRESIMS *m*/*z* 287.1250 [M + Na]^+^ (calcd. for C_15_H_20_NaO_4_, 287.1254).

Compound **5**: Yellow amorphous solid; [α${]}_{D}^{25}$ +1.8 (c 0.1, MeOH); UV (MeOH) λmax (log ε) 207(3.85) nm; IR (neat) *ν*_max_ 732, 765, 838, 883, 995, 1033, 1058, 1093, 1151, 1255, 1376, 1400, 1436, 1589, 1660, 1716, 1754, 2854, 2925, 2950, 3473 cm^-1^ (Figure S45); ^1^H NMR (CDCl_3_, 400 MHz) and ^13^C NMR (CDCl_3_, 100 MHz), see Table 3; HRESIMS *m*/*z* 359.18512 [M − H]^−^ (calcd. for C_21_H_27_O_5_, 358.1853).

Compound **6**: Yellow amorphous solid; [α${]}_{D}^{25}$ +44.95 (c 0.1, MeOH); UV (MeOH); CD (MeOH) *λ*max (Δ*ε*) 235 (−5.9); λmax (log ε) 365(3.47) nm; IR (neat) *ν*_max_ 729, 765, 881, 948, 997, 1029, 1066, 1128, 1155, 1261, 1324, 1380, 1434, 1714, 2854, 2923, 3398 cm^−1^ (Figure S54); ^1^H NMR (CDCl_3_, 400 MHz) and ^13^C NMR (CDCl_3_, 100 MHz), see Table 3; HRESIMS *m*/*z* 386.16132 [M − H]^−^ (calcd. for C_21_H_24_NO_6_, 387.16091).

### 3.4. Computational Detail

In optical rotation and ECD calculations, conformational analysis of compounds was performed using Spartan’14 software (Wavefunction Inc., Irvine, CA, USA) in the MMFF force field and the b3lyp/6-31+g(d,p) level was used to optimize the conformers, using this approach with the solvent model for MeOH [15,16]. Conformers with Boltzmann distributions over 5% were selected for ECD and OR calculations in methanol at the b3lyp/6-31+g(d,p) level. The ECD and OR spectra were generated using the programs SpecDis 1.71 (University of Würzburg, Würzburg, Germany) and OriginPro 2021 (OriginLab, Ltd., Northampton, MA, USA) based on dipole-length rotational strengths by applying Gaussian band shapes with sigma = 0.30 ev [17,18]. The optical rotations were calculated using time-dependent density functional theory. The b3lyp/6-31+g(d,p) level was employed in methanol for calculating optical rotations at A = 589.3 nm. All the calculations were performed using Tianhe-2 in the National Super Computer Center in Guangzhou.

### 3.5. Crystal Structure Analysis

Colorless crystals of compound **1** were obtained from a solvent of chloroform. Crystal data were acquired using the hemisphere technique on a Rigaku Oxford Diffraction diffractometer with graphite-monochromated Cu-K*α* (radiation λ = 1.54178 Å). The structure was solved by means of direct methods using SHELXT; refinement was carried out by full-matrix least squares on F2, using the SHELXT program suite on Olex2 Launcher [19,20].

Crystal Data of **1**—C_9_H_10_O_4_ (M = 182.17 g/mol): monoclinic, space group *P*2_1/c_ (no. 14), *a* = 6.4187(2) Å, *b* = 28.6008(9) Å, *c* = 4.7982(2) Å, *β* = 99.313(3)°, *V* = 869.24(5) Å^3^, *Z* = 4, *T* = 150.01(11) K, *μ*(Cu-K*α*) = 0.935 mm^−1^, *D_calc_* = 1.392 g/cm^3^, 3075 reflections measured (12.38° ≤ 2*θ* ≤ 147.79°), 1688 unique (*R*_int_ = 0.0306, *R*_sigma_ = 0.0447), which were used in all calculations. The final *R*_1_ was 0.0439 (I > 2*σ*(I)) and *wR*_2_ was 0.1396 (all data). Crystallographic data for **1** have been deposited in the Cambridge Crystallographic Data Center as supplementary publication number CCDC 1989670.

### 3.6. Anti-Inflammatory Activity

Evaluation of their inhibition of nitric oxide (NO) production in RAW264.7 cells activated by lipopolysaccharide (LPS) was carried out using the Griess assay with indomethacin as a positive control, according to a previous report [21]. RAW264.7 cells were purchased from the cell bank of the Chinese Academy of Sciences.

## 4. Conclusions

In this study, a chemical investigation on the secondary metabolites of *Fusarium solani* 7227, isolated from seawater in the South China Sea, led to the isolation of compounds **1**–**11**. Among the isolates, compounds **1** and **2** were found to be possible precursor compounds and compounds **3**–**6** were a series of new fusarin-related compounds with a polyunsaturated side chain. Together with the known compounds **7**–**11**, all compounds were evaluated for their inhibition of NO production in RAW264.7 cells activated by LPS. Compounds **2**–**5** and **7**–**11** displayed potent anti-inflammatory activity, with IC_50_ values ranging from 3.6 to 32.2 μM. A preliminary structure–activity relationship analysis indicated that the anti-inflammatory activities depend on the double bonds between C-4 and C-5 for compounds **1** and **2** and on the substitution group of the polyunsaturated chain for compounds **3**–**11**.

## Figures and Tables

**Table 1:** ^1^H NMR (400 MHz) and ^13^C NMR (100 MHz) data for **1** and **2** in CDCl_3_.

No.	1		2	
	*δ*_C_ Type	*δ*_H_ (*J* in Hz)	*δ*_C_ Type	*δ*_H_ (*J* in Hz)
1	16.0 CH_3_	1.78 (d, 1.4)	14.7 CH_3_	1.82 (d, 7.2)
2	142.2 CH	7.08 (qd, 7.2, 1.1)	140.3 CH	6.9 (q, 7.2)
3	129.3 C		130.2	
4	135.3 CH	7.36 (m)	30.1 CH_2_	2.72 (m, overlap)
4′				2.4 (m)
5	131.0 C		38.9 CH	2.72 (m, overlap)
6	172.8 C		182.4	
7	166.7 C		168.1	
8	52.2 OCH_3_	3.76 (s)	51.9 OCH_3_	3.73 (s)
9	14.3 CH_3_	1.76 (d, 1.4)	16.7 CH_3_	1.16 (d, 6.6)

**Table 2:** ^1^H NMR (400 MHz) and ^13^C NMR (100 MHz) data for fusarins I (**3**) and J (**4**) in CDCl_3_.

No.	3		4	
	*δ*_C_ Type	*δ*_H_ (*J* in Hz)	*δ*_C_ Type	*δ*_H_ (*J* in Hz)
1	16.0 CH_3_	1.74 (d 7.3)	14.6 CH_3_	1.92 (d 7.2)
2	140.5 CH	6.98 (q 7.1)	141.1 CH	7.05 (q 7.2)
3	130.5		131.8	
4	127.1 CH	6.18 (s)	71.8 CH	5.06 (d 10.5)
5	138.1		144.2	
6	140.6 CH	6.58 (m)	123.7 CH	6.28 (d 11.4)
7	128.6 CH	6.43 (dd 15.2, 10.3)	137.5 CH	6.86 (dd 14.7, 11.4)
8	140.6 CH	6.66(dd 14.6, 10.4)	130.6 CH	6.34 (dd 14.7, 11.2)
9	127.9 CH	6.55 (d 14.7)	143.9 CH	7.20 (dd 15.4, 11.1)
10	140.9 CH	7.37 (d 11.3)	129.8 CH	6.13 (d 15.4)
11	126.0		198.6	
12	173.6		27.5 CH_3_	2.27 (s)
13	167.7		167.8	
14	52.1 OCH_3_	3.74 (s)	52.1 OCH_3_	3.74 (s)
15	14.4 CH_3_	1.70 (s)	14.5 CH_3_	1.80 (d 1.3)
16	12.5 CH_3_	1.98 (s)		

**Table 3:** ^1^H NMR (400 MHz) and ^13^C NMR (100 MHz) data for fusarins K (**5**) and L (**6**) in CDCl_3_.

No.	5		6	
	*δ*_C_ Type	*δ*_H_ (*J* in Hz)	*δ*_C_ Type	*δ*_H_ (*J* in Hz)
1	16.1 CH_3_	1.75 (dd 7.2, 1.4)	16.1 CH_3_	1.73 (dd 7.2, 1.3)
2	140.6 CH	6.99 (q 7.5)	140.6 CH	6.97 (m)
3	130.5		130.3	
4	127.5 CH	6.20 (s)	128.1 CH	6.2 (s)
5	138.2 C		138.2	
6	141.1 CH	6.59 (m)	142.4 CH	6.60 (m)
7	128.6 CH	6.4 (dd 15.2, 9.7)	128.6 CH	6.43 (dd 15.2, 10.9)
8	141.05 CH	6.56 (m)	143.9	6.80 (dd 14.6, 10.8)
9	128.4 CH	6.63 (m)	128.2 CH	6.64 (m)
10	139.7 CH	7.09 (d 8.9)	146.0 CH	7.50 (d 11.4)
11	135.3		133.8	
12	196.9		190.5	
13	75.9 CH	4.06 (dd 5.3, 3.5)	62.7	
14	39.72 CH_2_	3.13 (dd 17.5, 5.4)	64.1 CH	3.94 (d 2.4)
		3.37 (dd 17.5 3.5)		
15	216.2		83.9	
16	36.7 CH_2_	2.54 (ddd 17.9, 7.6, 4.3)	170.6	
		2.87 (dt 17.9, 8.9)		
17	65.3 CH_2_	4.13 (q 7.9)	21.8 CH_3_	1.55 (s)
		4.37 (td 9.1, 4.3)		
18	167.7		167.6	
19	52.1 OCH_3_	3.75 (s)	52.1	5.28 (s)
20	14.4 CH_3_	1.71 (d 1.2)	14.3 CH_3_	1.68 (s)
21	11.8 CH_3_	1.91 (d 1.2)	11.5	1.92 (s)

**Table 4:** Inhibitory activity of all compounds **1**–**11** against lipopolysaccharide (LPS)-induced NO production in the murine macrophage cell line (RAW 264.7 cells).

Compounds	IC_50_ (μM)
**1**	>50
**2**	22.3 ± 5.0
**3**	13.1 ± 6.8
**4**	13.9 ± 7.5
**5**	21.9 ± 9.8
**6**	>50
**7**	32.2 ± 5.7
**8**	17.8 ± 4.9
**9**	7.6 ± 2.0
**10**	3.6 ± 2.2
**11**	8.4 ± 2.2
Indometacin ^a^	32.1 ± 2.5

^a^ Positive control.

## Data Availability

Data is contained within the article or supplementary material.

## Supplementary Materials

The following are available online at https://www.mdpi.com/article/10.3390/md19060305/s1, Figures S1–S54: ^1^H NMR, ^13^C NMR, DEPT, HSQC, HMBC, COSY, IR, and HRESIMS spectra of compounds **1**−**6**. Figures S55–S59: The optimized low energy conformers of compounds **2**, **4**, **5,** and **6**. Tables S1–S5 Energy analysis for the conformers of compounds (5*S*)-**2**, (4*S*)-**4**, (13*S*)-**5**, and (13*R*, 14*R*, 15*S*)-**6** and (13*R*, 14*R*, 15*R*)-**6.**
