# Supplementary material for: Fusarins G–L with Inhibition of NO in RAW264.7 from Marine-Derived Fungus Fusarium solani 7227
Source: Mar Drugs. 2021 May 25;19(6):305. doi: 10.3390/md19060305 (PMC8226964; doi:10.3390/md19060305)
Supplement: Supplementary file 1 [file marinedrugs-19-00305-s001.zip › marinedrugs-1217741-supplementary.pdf]

Fusarins G-L with inhibition of NO from marine-derived fungus *Fusarium solani* 7227

Guangyuan luo [1, †], Li Zheng [1, †], Qilin Wu [1] , Shenhua Chen [1] , Jing Li [1, 3,*] and Lan Liu [1, 2, 4]

[1] School of Marine Sciences, Sun Yat-Sen University, Guangzhou 510006, P. R. China; luogy5@mail2.sysu.edu.cn; zhengli26@mail2.sysu.edu.cn; wuqlin3@mail2.sysu.edu.cn; chensenh@mail.sysu.edu.cn; lijing356@mail.sysu.edu.cn; cesllan@mail.sysu.edu.cn;

[2] Southern Marine Science and Engineering Guangdong Laboratory (Zhuhai), Zhuhai 519000, P. R. China;

[3] Guangdong Provincial Key Laboratory of Marine Resources and Coastal Engineering, Zhuhai, China, 519082

[4] Pearl River Estuary Marine Ecosystem Research Station, Ministry of Education, Zhuhai, 519082, China

* Correspondence: lijing356@mail.sysu.edu.cn;

† These authors contributed equally to this work.

Supplementary Information

Figure S 1. ^{1}H NMR spectrum of 1 in $CDCl_3$. 5
Figure S 2. ^{13}C NMR spectrum of 1 in $CDCl_3$. 5
Figure S 3. DEPT-90 spectrum of 1 in $CDCl_3$. 6
Figure S 4. DEPT-135 spectrum of 1 in $CDCl_3$. 6
Figure S 5. HSQC spectrum of 1 in $CDCl_3$. 7
Figure S 6. HMBC spectrum of 1 in $CDCl_3$. 7
Figure S 7. ^{1}H-^{1}H COSY spectrum of 1 in $CDCl_3$. 8
Figure S 8. HR-ESIMS spectrum of 1. 9
Figure S 9. IR spectrum of 1. 10
Figure S 10. ^{1}H NMR spectrum of 2 in $CDCl_3$. 11
Figure S 11. ^{13}C NMR spectrum of 2 in $CDCl_3$. 11
Figure S 12. DEPT-90 spectrum of 2 in $CDCl_3$. 12
Figure S 13. DEPT-135 spectrum of 2 in $CDCl_3$. 12
Figure S 14. HSQC spectrum of 2 in $CDCl_3$. 13
Figure S 15. HMBC spectrum of 2 in $CDCl_3$. 13
Figure S 16. ^{1}H-^{1}H COSY spectrum of 2 in $CDCl_3$. 14
Figure S 17. HR-ESIMS spectrum of 2.. 14
Figure S 18. IR spectrum of 2. 15
Figure S 19. ^{1}H NMR spectrum of 3 in $CDCl_3$. 15
Figure S 20. ^{13}C NMR spectrum of 3 in $CDCl_3$. 16
Figure S 21. DEPT-90 spectrum of 3 in $CDCl_3$. 16
Figure S 22. DEPT-135 spectrum of 3 in $CDCl_3$. 17
Figure S 23. HSQC spectrum of 3 in $CDCl_3$. 17
Figure S 24. HMBC spectrum of 3 in $CDCl_3$. 18
Figure S 25. ^{1}H-^{1}H COSY spectrum of 3 in $CDCl_3$. 18
Figure S 26. HR-ESIMS spectrum of 3. 19
Figure S 27. IR spectrum of 3. 19
Figure S 28. ^{1}H NMR spectrum of 4 in $CDCl_3$. 20

Figure S 29. ^{13}C NMR spectrum of 4 in $CDCl_3$. 20
Figure S 30. DEPT-90 spectrum of 4 in $CDCl_3$. 21
Figure S 31. DEPT-135 spectrum of 4 in $CDCl_3$. 21
Figure S 32. HSQC spectrum of 4 in $CDCl_3$. 22
Figure S 33. HMBC spectrum of 4 in $CDCl_3$. 22
Figure S 34. ^{1}H-^{1}H COSY spectrum of 4 in $CDCl_3$. 23
Figure S 35. HR-ESIMS spectrum of 4. 24
Figure S 36. IR spectrum of 4. 25
Figure S 37. ^{1}H NMR spectrum of 5 in $CDCl_3$. 25
Figure S 38. ^{13}C NMR spectrum of 5 in $CDCl_3$. 26
Figure S 39. DEPT-90 spectrum of 5 in $CDCl_3$. 26
Figure S 40. DEPT-135 spectrum of 5 in $CDCl_3$. 27
Figure S 41. HSQC spectrum of 5 in $CDCl_3$. 27
Figure S 42. HMBC spectrum of 5 in $CDCl_3$. 28
Figure S 43. ^{1}H-^{1}H COSY spectrum of 5 in $CDCl_3$. 28
Figure S 45. IR spectrum of 5. 29
Figure S 46. ^{1}H NMR spectrum of 6 in $CDCl_3$. 30
Figure S 47. ^{13}C NMR spectrum of 6 in $CDCl_3$ 30
Figure S 48. DEPT-90 spectrum of 6 in $CDCl_3$. 31
Figure S 49. DEPT-135 spectrum of 6 in $CDCl_3$. 31
Figure S 50. HSQC spectrum of 6 in $CDCl_3$. 32
Figure S 51. HMBC spectrum of 6 in $CDCl_3$. 32
Figure S 52. ^{1}H-^{1}H COSY spectrum of 6 in $CDCl_3$. 33
Figure S 53. HR-ESIMS spectrum of 6. 33
Figure S 54. IR spectrum of 6. 34
Table S1. Energy Analysis for the Conformers of (5*S*)-2. 35
Figure S 55. The optimized low energy conformers of 2. 35
Table S2. Energy Analysis for the Conformers of (4*S*)-4. 36
Figure S 56. The optimized low energy conformers of 4. 37
Table S3. Energy Analysis for the Conformers of (13*S*)-5. 38

Figure S 57. The optimized low energy conformers of 5. 39

Table S4. Energy Analysis for the Conformers of (13*R*, 14*R*,15*S*)-6. 39

Figure S 58. The optimized low energy conformers of (13*R*, 14*R*,15*S*)-6. 40

Table S5. Energy Analysis for the Conformers of (13*R*, 14*R*,15*R*)-6. 40

Figure S 59. The optimized low energy conformers of (13*R*, 14*R*,15*R*)-6. 41

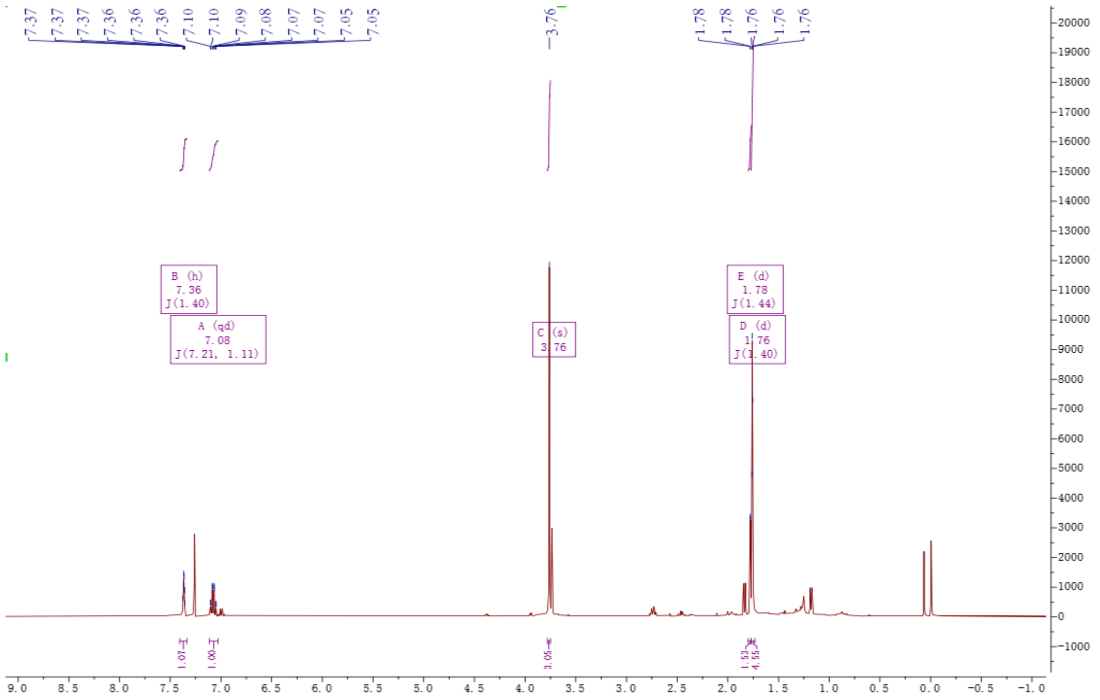

Figure S 1. ^{1}**H NMR spectrum of 1 in** $CDCl_3$**.**

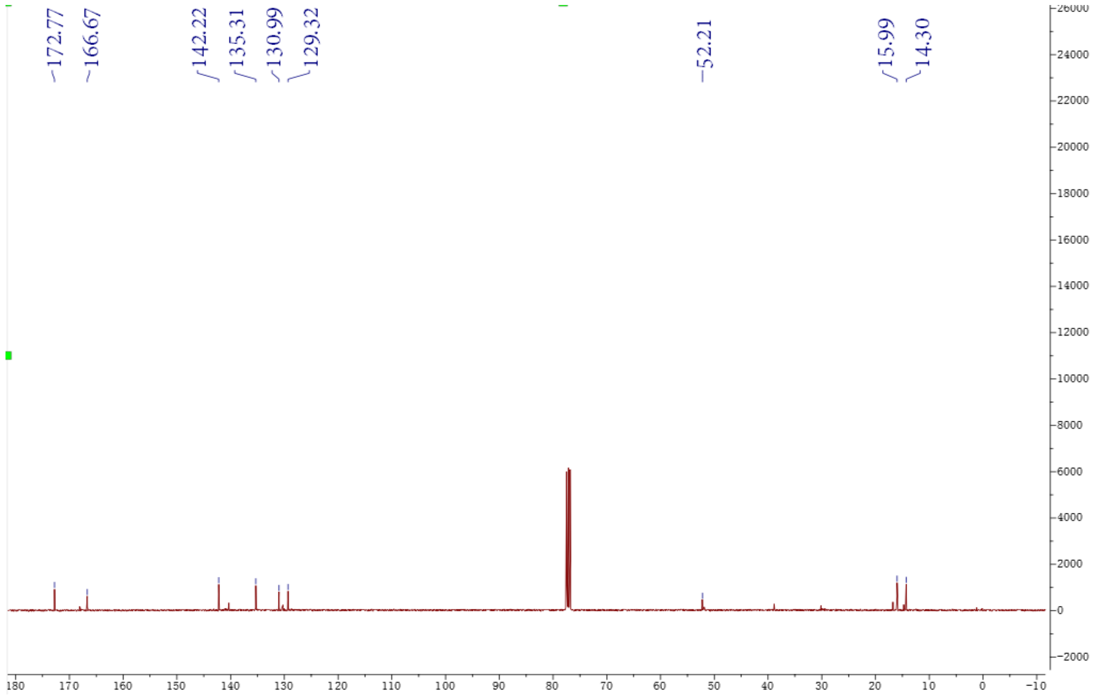

Figure S 2. ^{13}**C NMR spectrum of 1 in** $CDCl_3$**.**

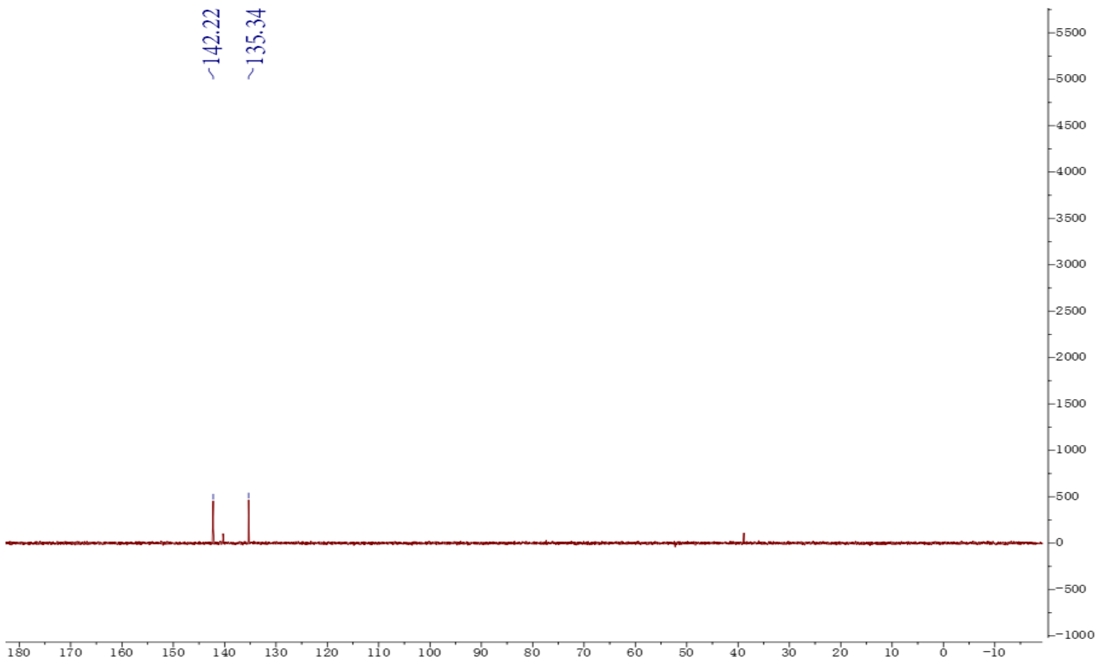

Figure S 3. DEPT-90 spectrum of 1 in $CDCl_3$.

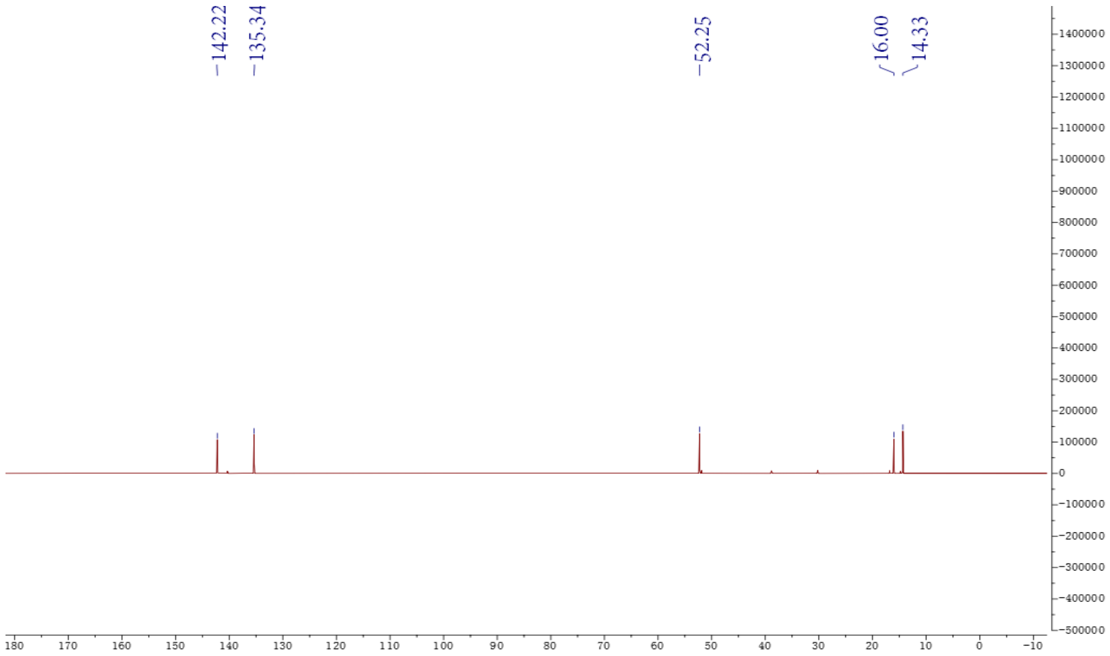

Figure S 4. DEPT-135 spectrum of 1 in $CDCl_3$.

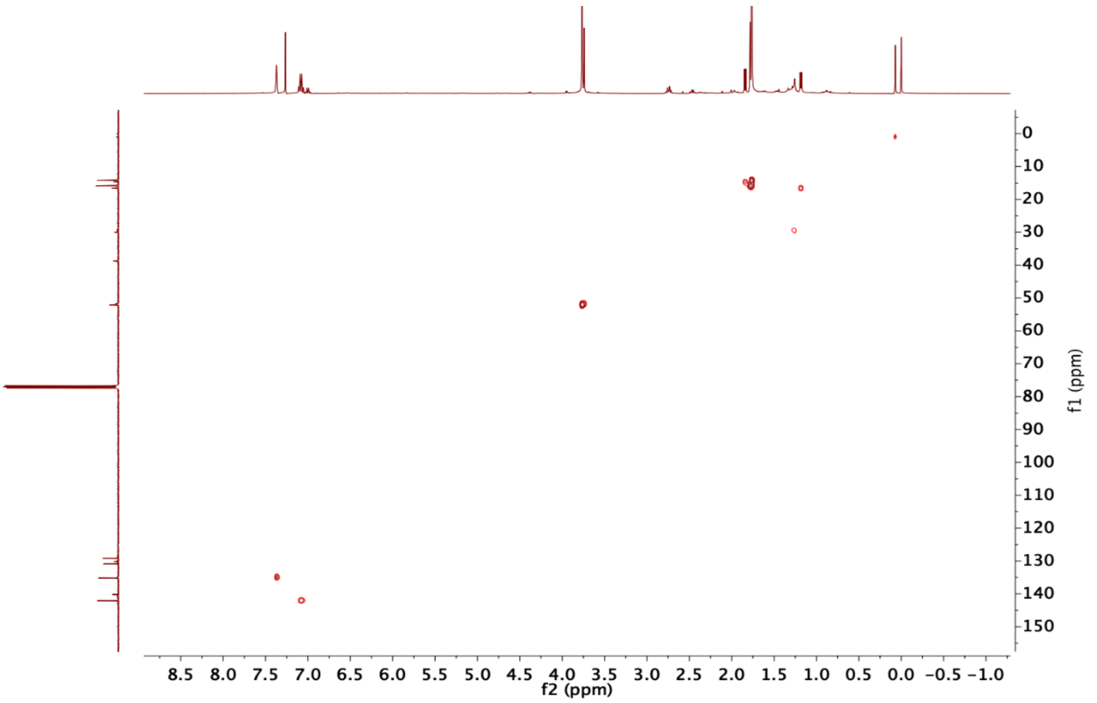

Figure S 5. HSQC spectrum of 1 in $CDCl_3$.

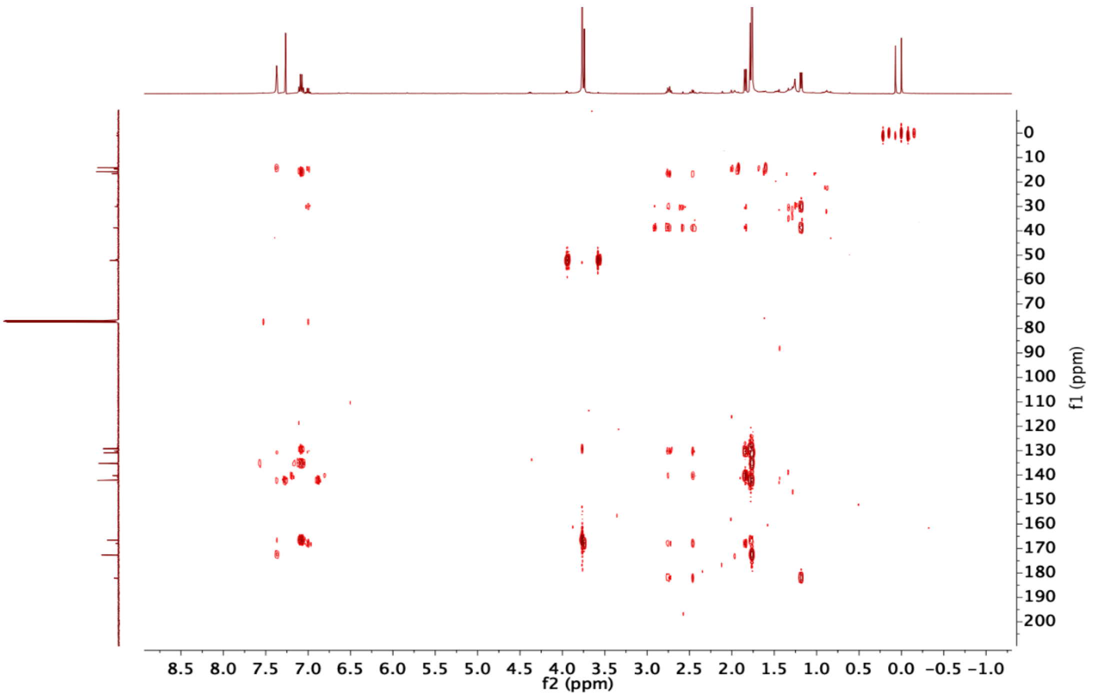

Figure S 6. HMBC spectrum of 1 in $CDCl_3$.

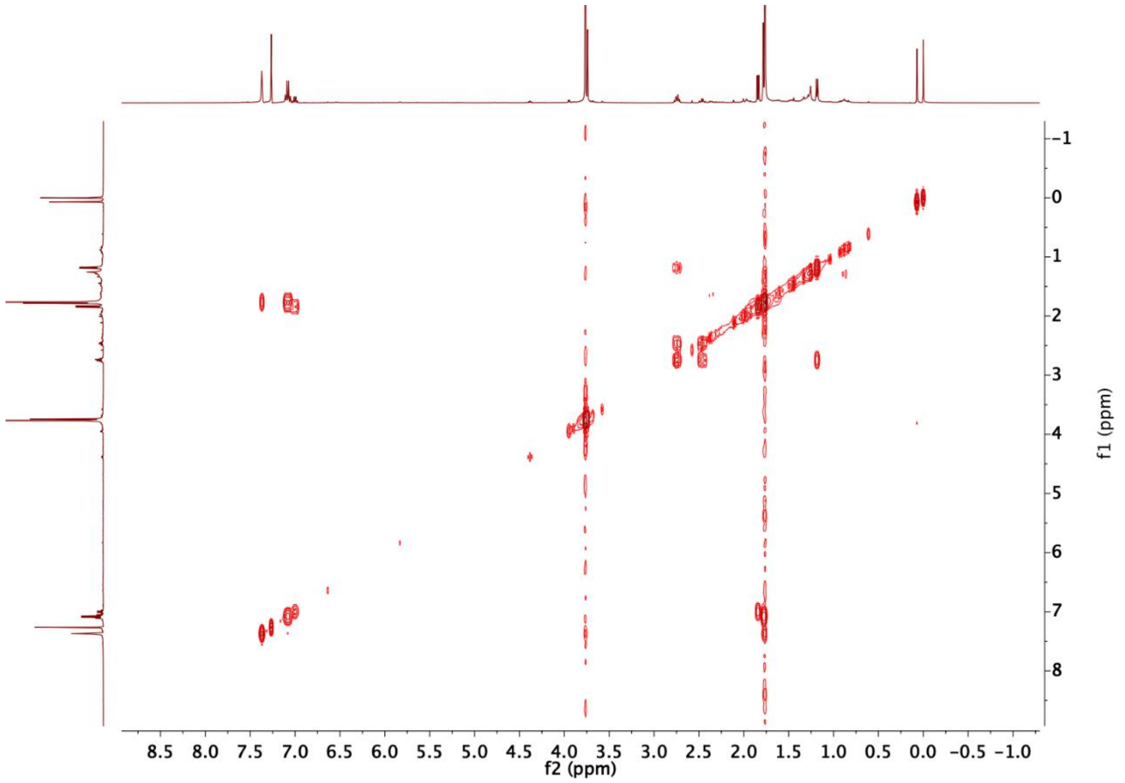

Figure S 7. ^{1}H-^{1}H **COSY spectrum of 1 in** $CDCl_3$.

Analysis Info

Analysis Name	D:\Data\2020\05\Liu lan\2005A0279\2005A0279_7227-15_neg_1uL_RA3_01_3541.d	Acquisition Date	5/22/2020 11:32:29 AM
Method	Tune_neg_low_LCMS.m	Operator	Demo User
Sample Name	2005A0279_7227-15_neg_1uL	Instrument	timsTOF 1844426.00062
Comment			

Acquisition Parameter

Source Type	ESI	Ion Polarity	Negative	Set Nebulizer	0.6 Bar
Focus	Not active	Set Capillary	3500 V	Set Dry Heater	200 °C
Scan Begin	100 m/z	Set End Plate Offset	-500 V	Set Dry Gas	6.0 l/min
Scan End	1000 m/z	Set Collision Cell RF	500.0 Vpp	Set Divert Valve	Waste

-MS, 0.08-0.09min

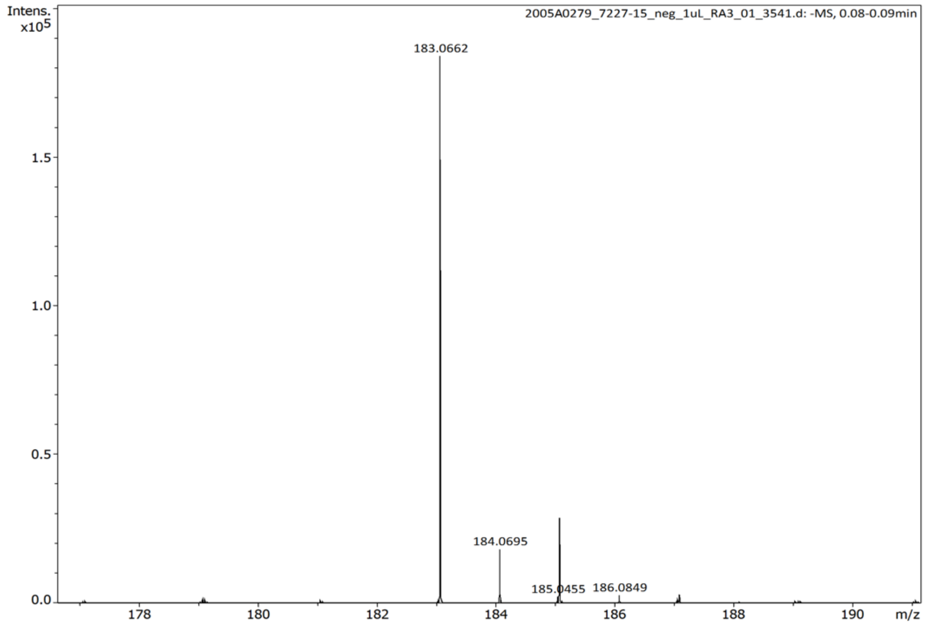

Meas. m/z	Ion Formula	m/z	z	err [ppm]	err [mDa]	mSigma	rdb	e⁻ Conf	N-Rule
183.0662	C9H11O4	183.0663	1-	0.3	0.1	7.4	4.0	even	ok
	C5H7N6O2	183.0636	1-	-14.3	-2.6	12.6	5.0	even	ok
	C10H7N4	183.0676	1-	7.6	1.4	15.2	9.0	even	ok
	C4H11N2O6	183.0623	1-	-21.6	-4.0	26.7	0.0	even	ok

Figure S 8. HR-ESIMS spectrum of 1.

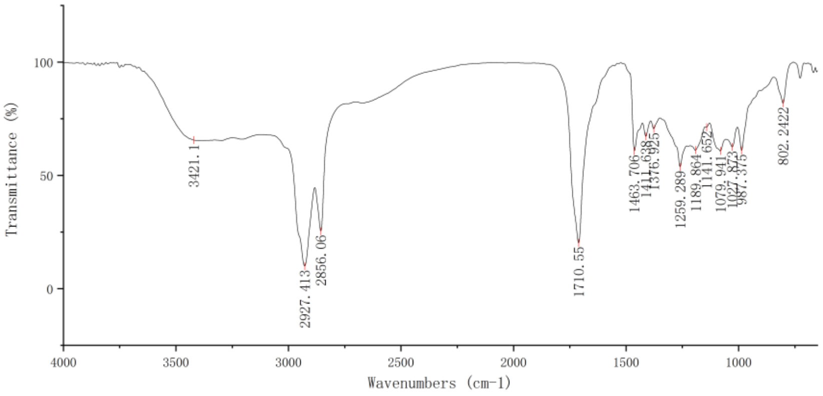

Figure S 9. IR spectrum of 1.

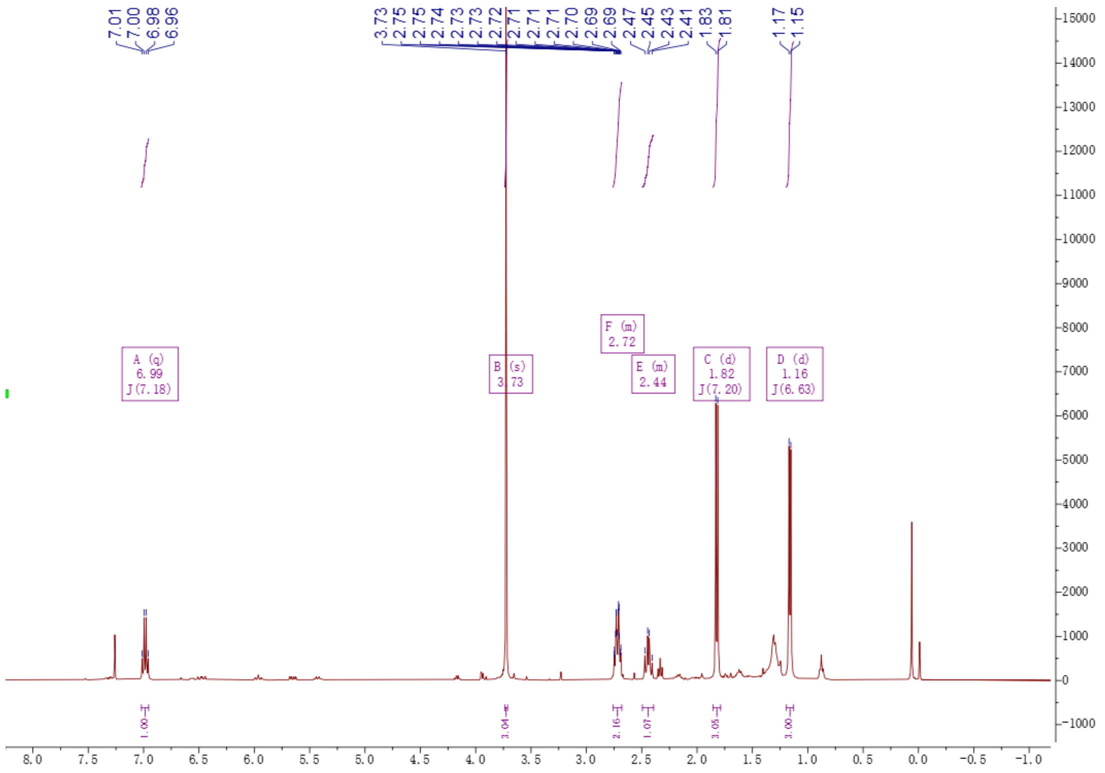

Figure S 10. [1]**H NMR spectrum of 2 in CDCl**$_3$**.**

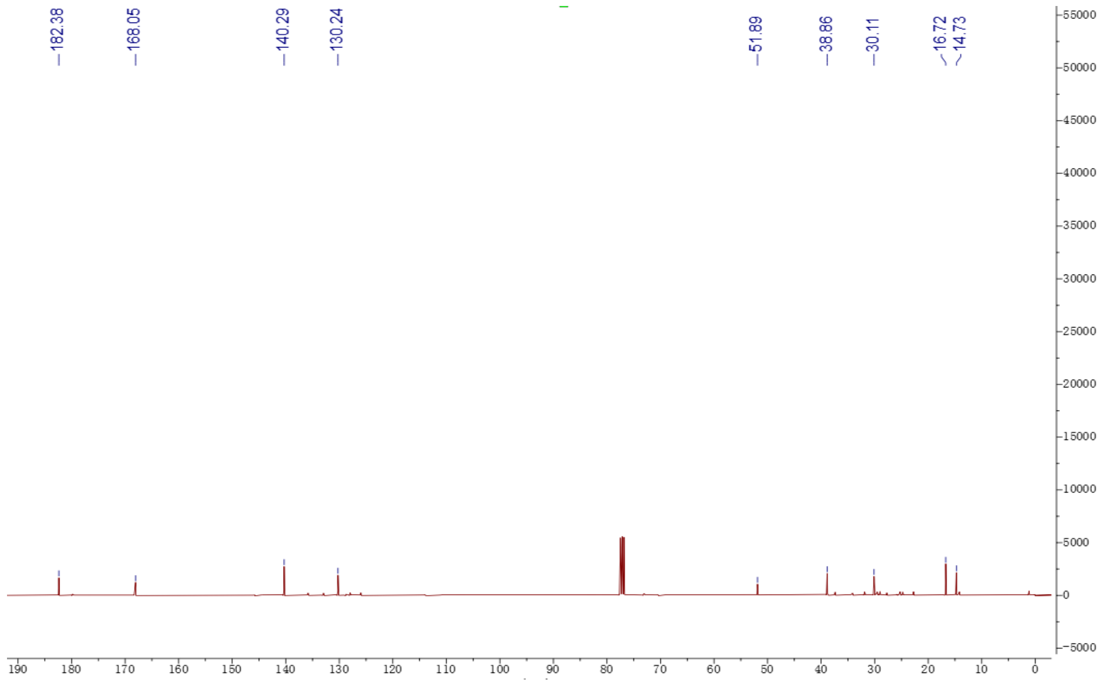

Figure S 11. [13]**C NMR spectrum of 2 in CDCl**$_3$**.**

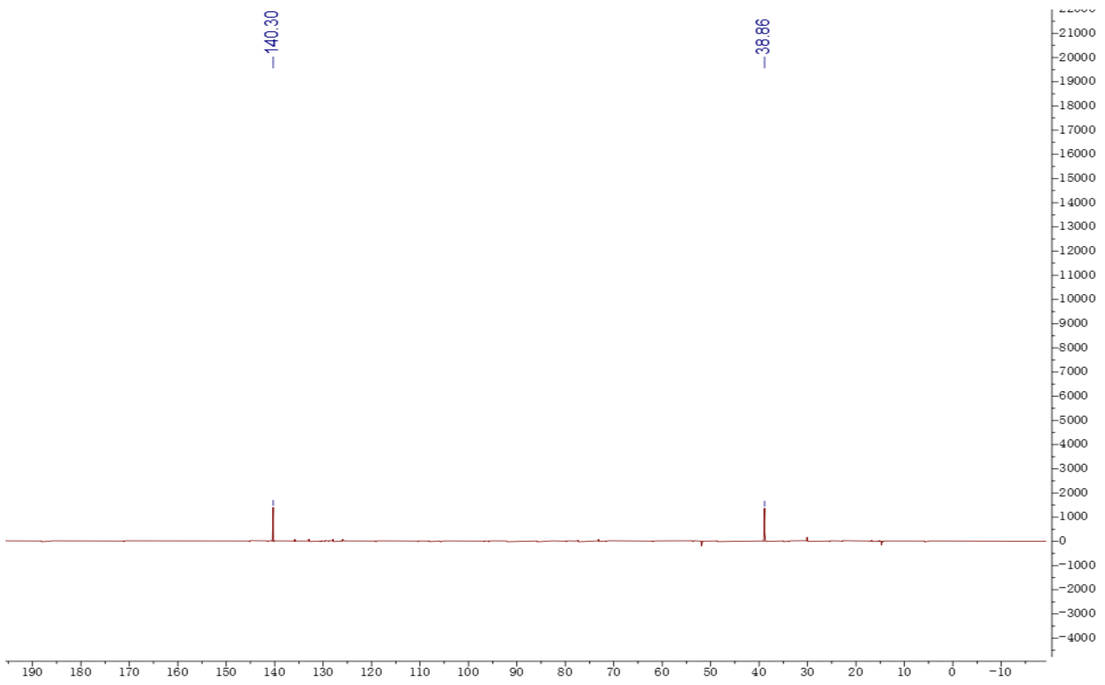

Figure S 12. DEPT-90 spectrum of 2 in $CDCl_3$.

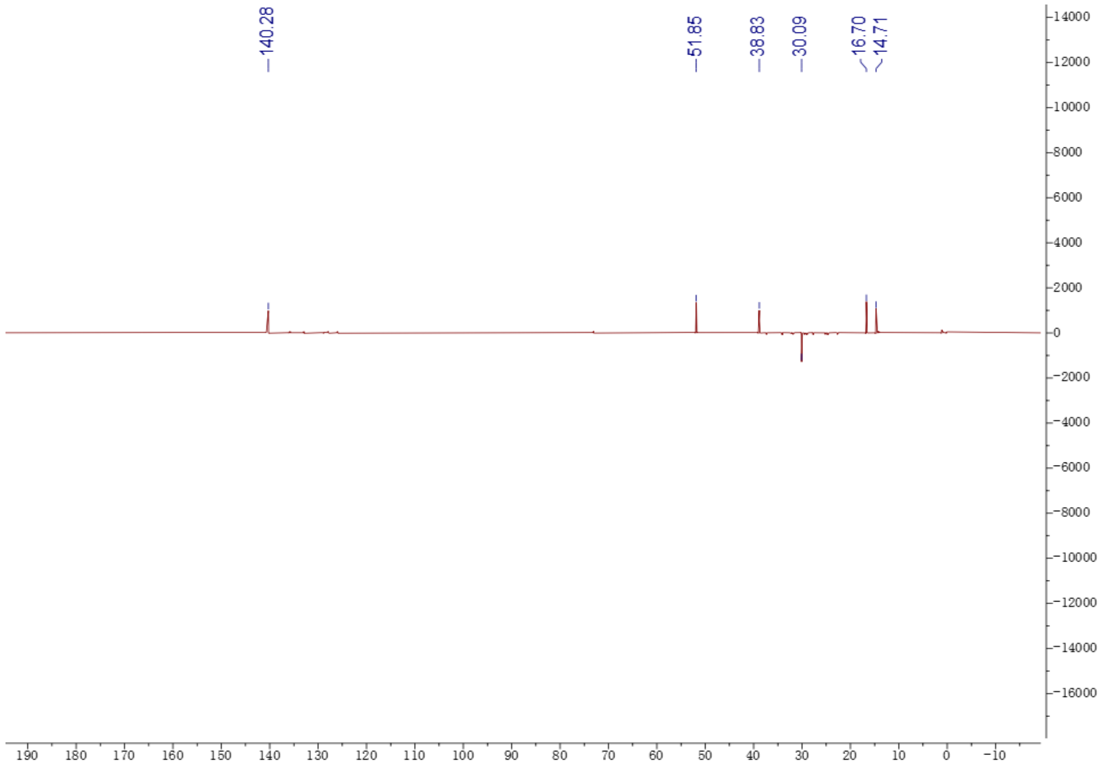

Figure S 13. DEPT-135 spectrum of 2 in $CDCl_3$.

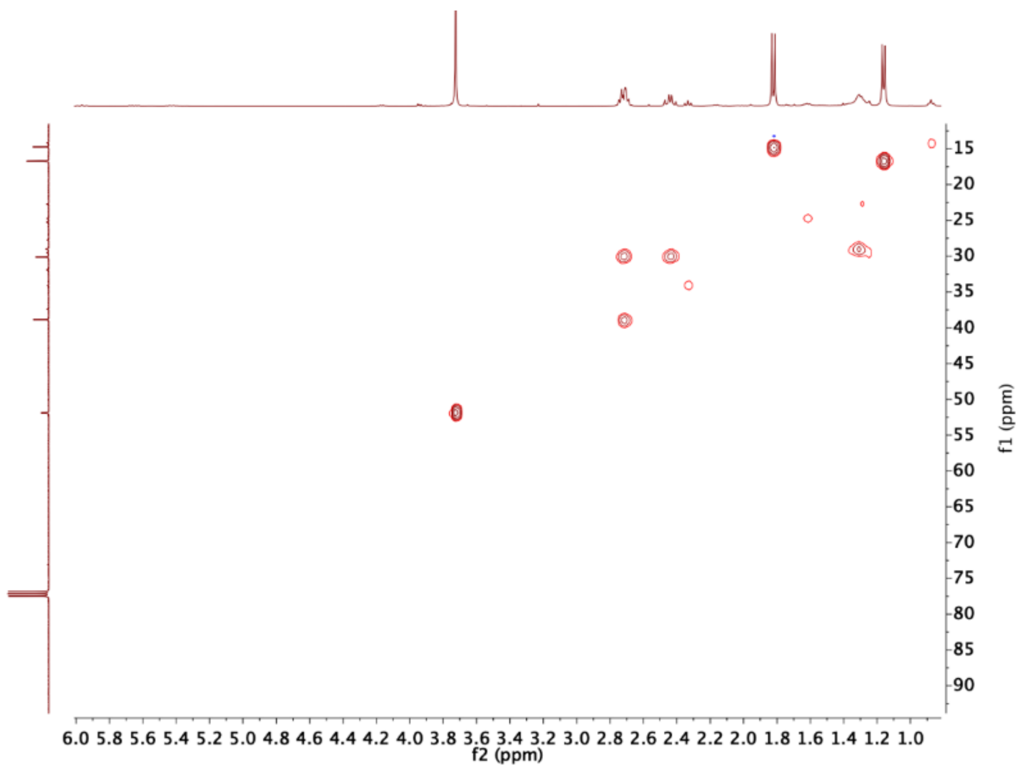

Figure S 14. HSQC spectrum of 2 in CDCl3.

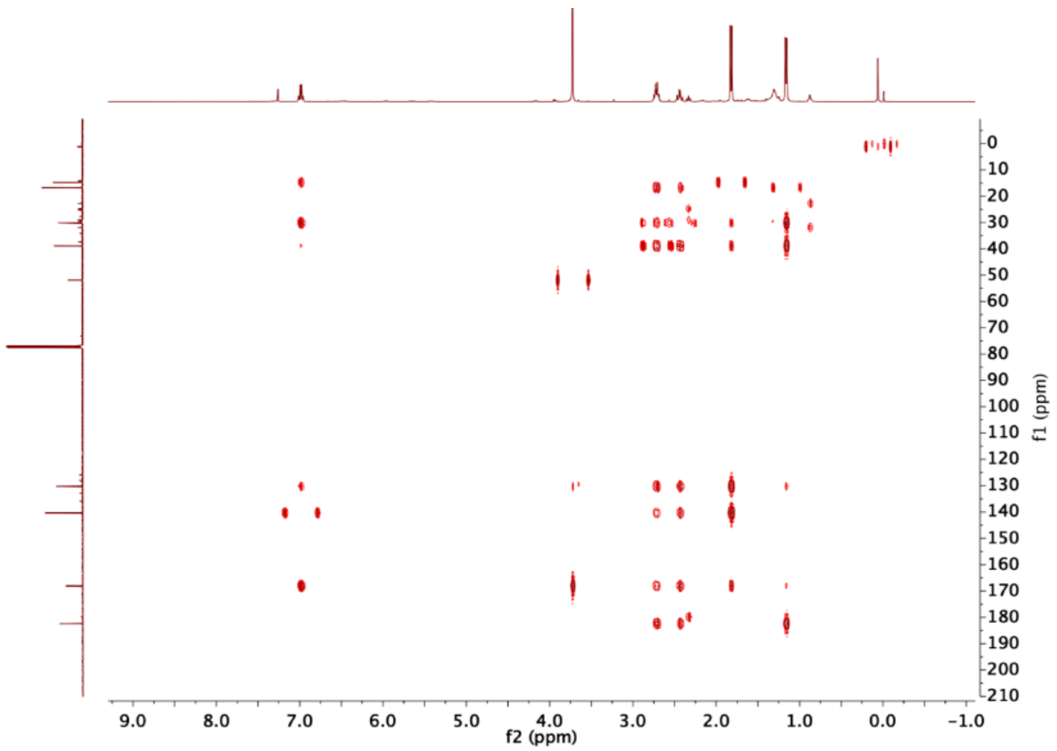

Figure S 15. HMBC spectrum of 2 in CDCl3.

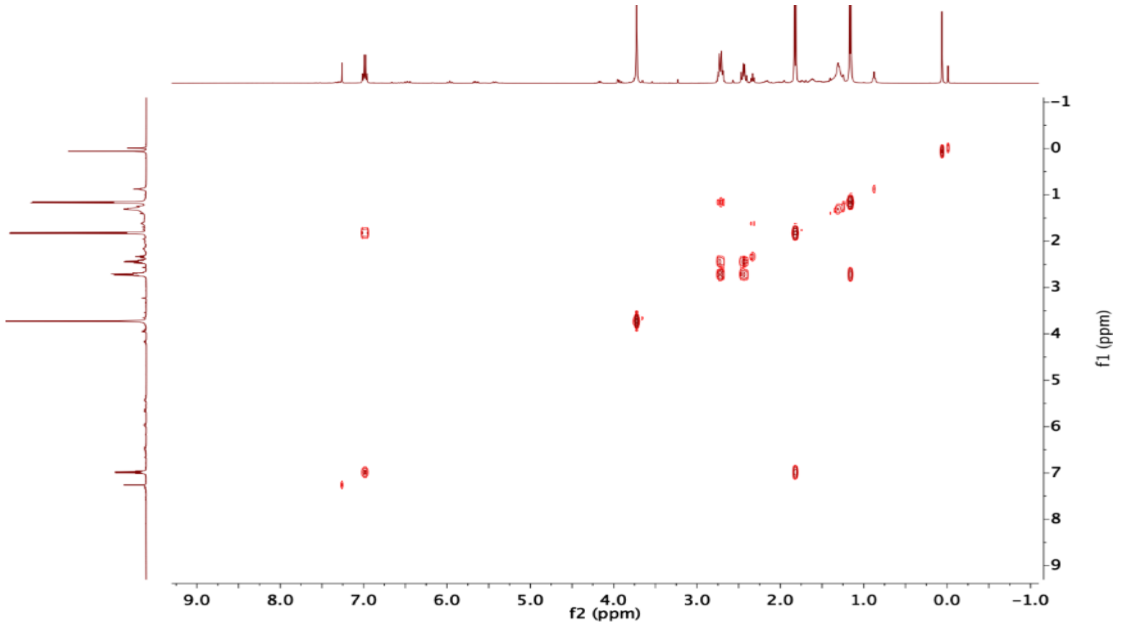

Figure S 16. ^{1}H-^{1}H COSY spectrum of 2 in $CDCl_3$.

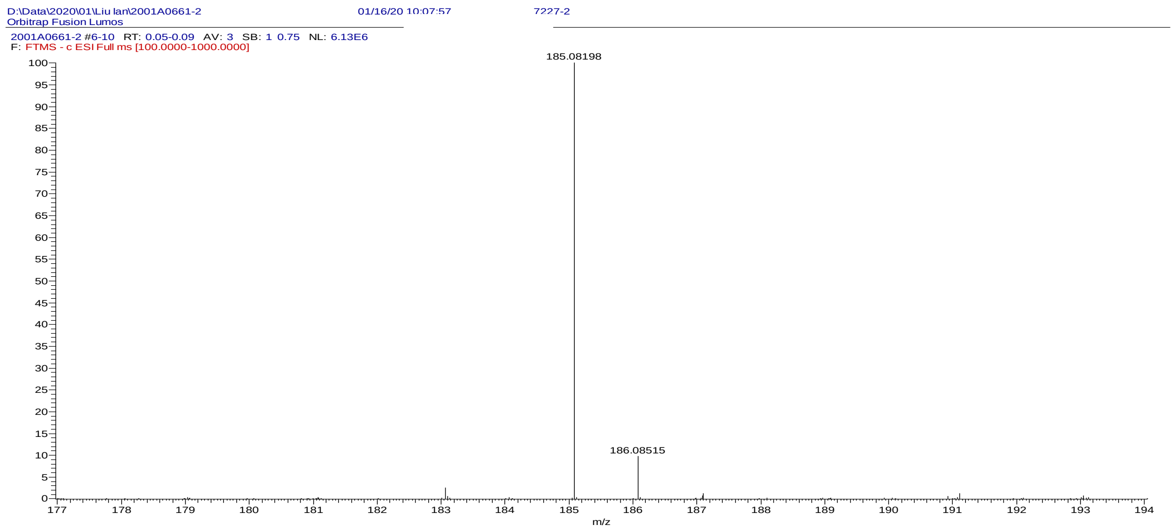

SPECTRUM - simulation : m/z	Theo. Mass	Delta (ppm)	RDB equiv.	Composition
185.08198	185.08193	0.26	3.5	C9 H13 O4

Figure S 17. HR-ESIMS spectrum of 2..

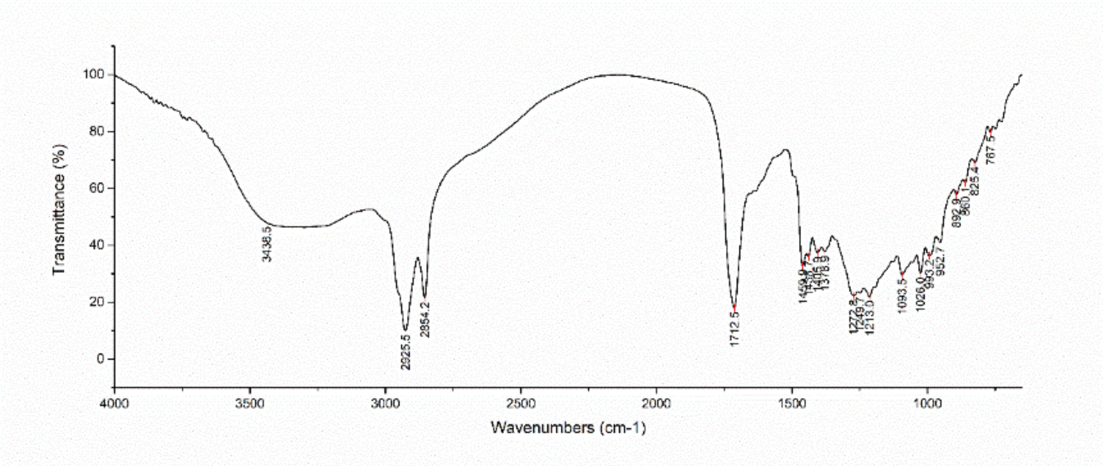

Figure S 18. IR spectrum of 2.

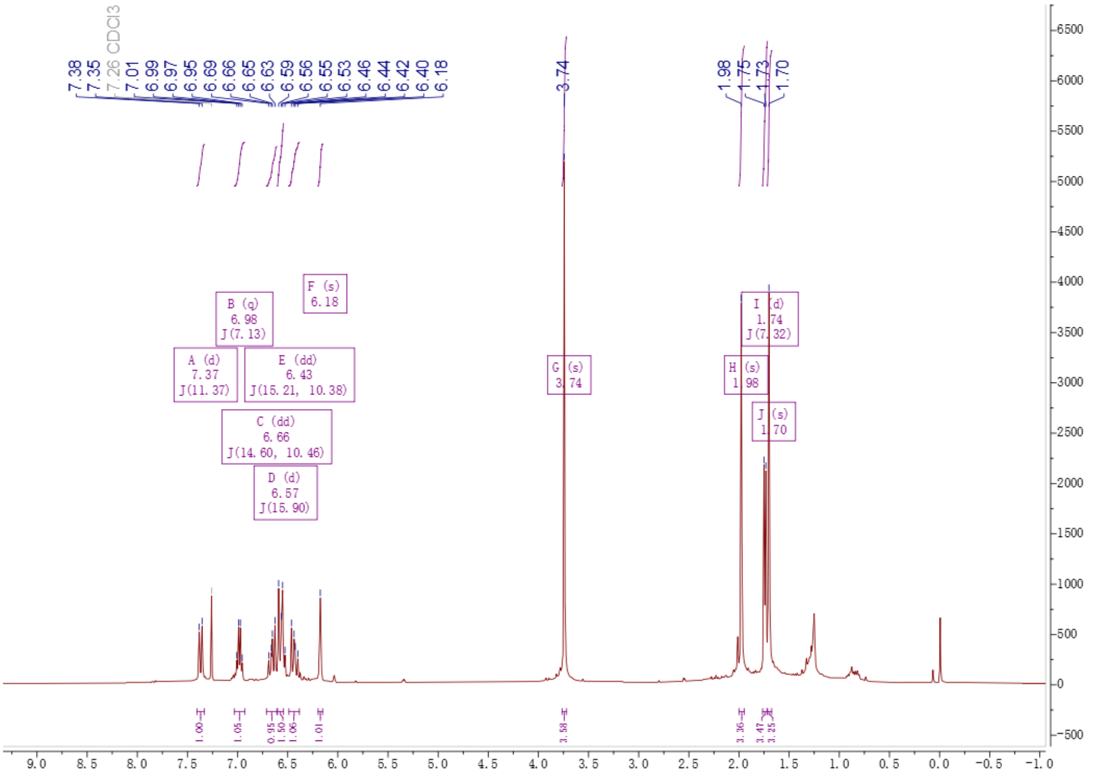

Figure S 19. ^{1}H NMR spectrum of 3 in $CDCl_3$.

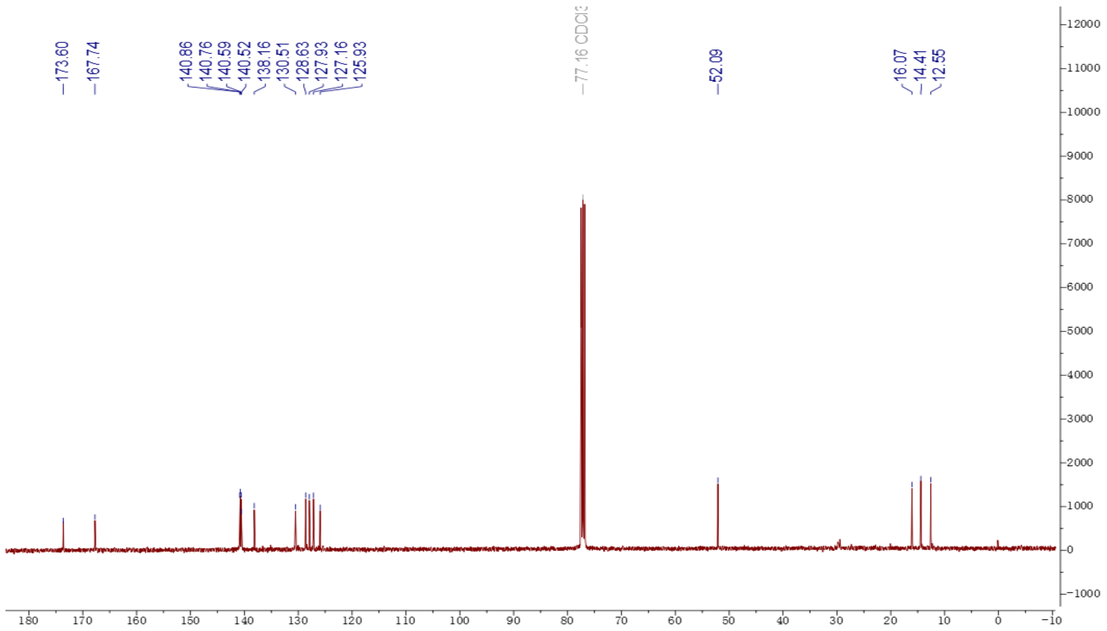

Figure S 20. ^{13}C NMR spectrum of 3 in CDCl$_3$.

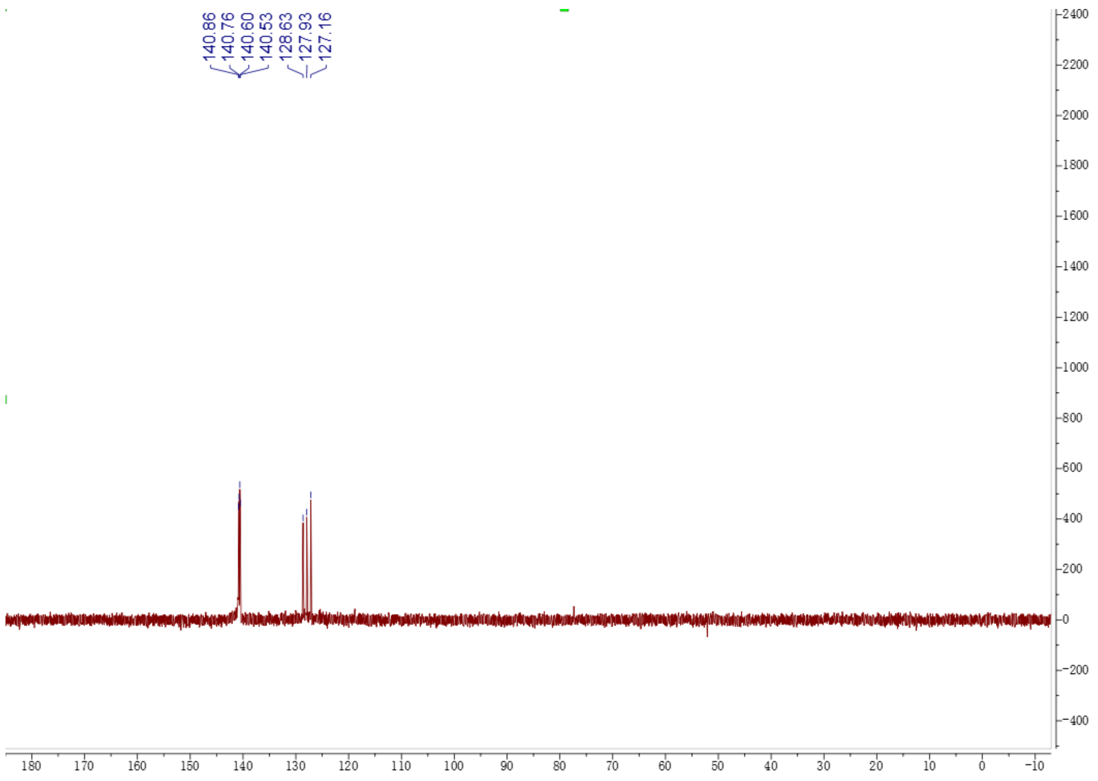

Figure S 21. DEPT-90 spectrum of 3 in CDCl$_3$.

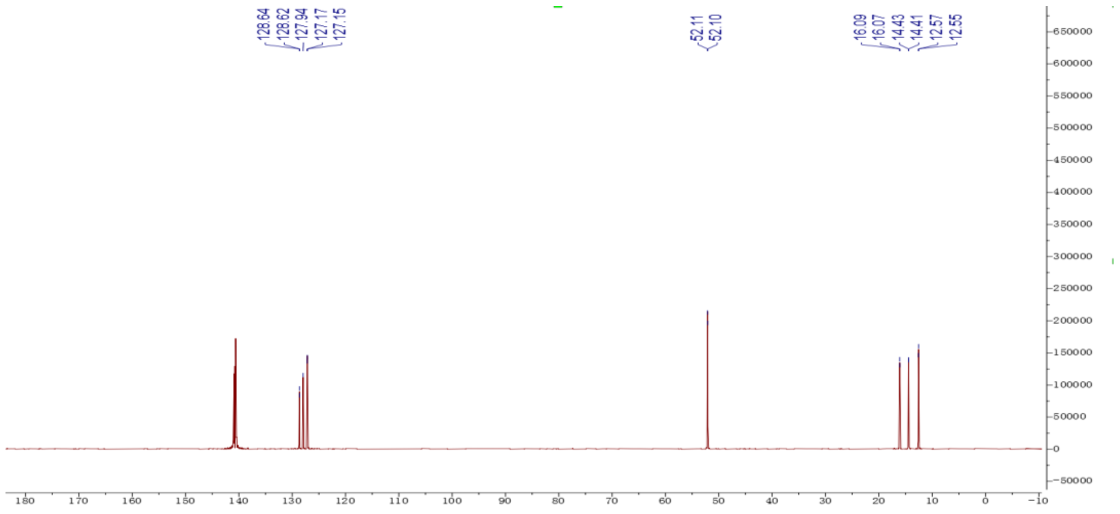

Figure S 22. DEPT-135 spectrum of 3 in CDCl3.

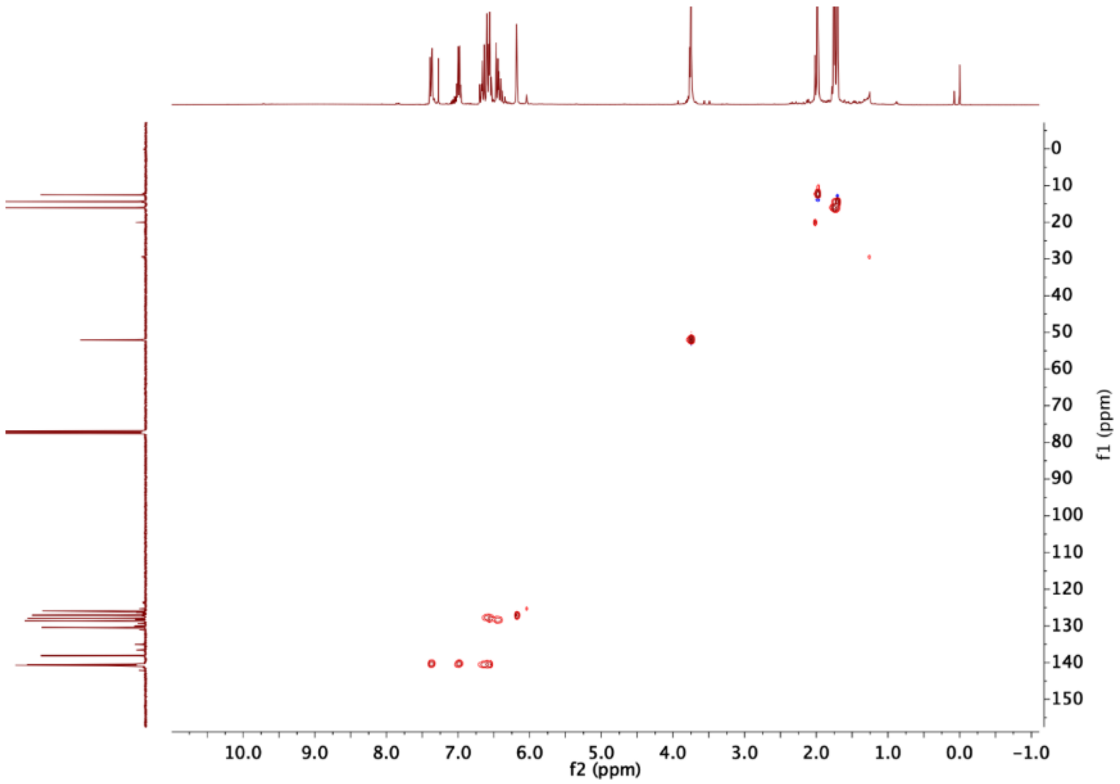

Figure S 23. HSQC spectrum of 3 in CDCl3.

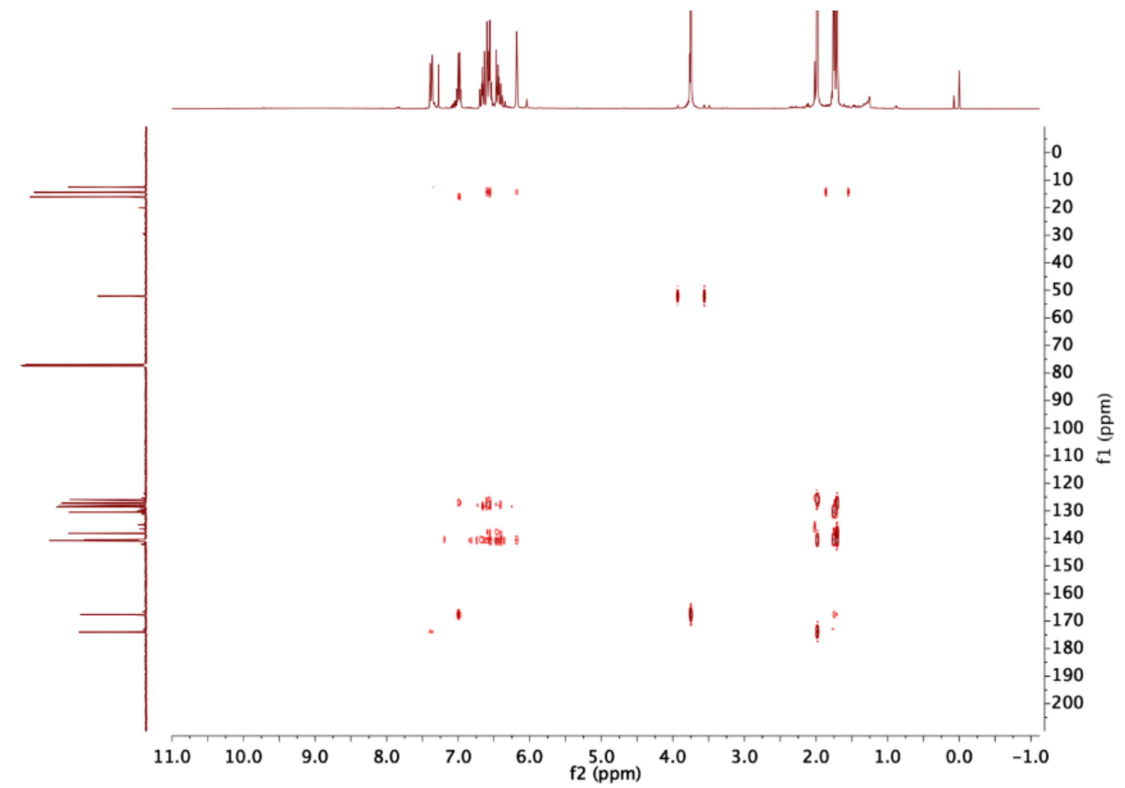

Figure S 24. HMBC spectrum of 3 in $CDCl_3$.

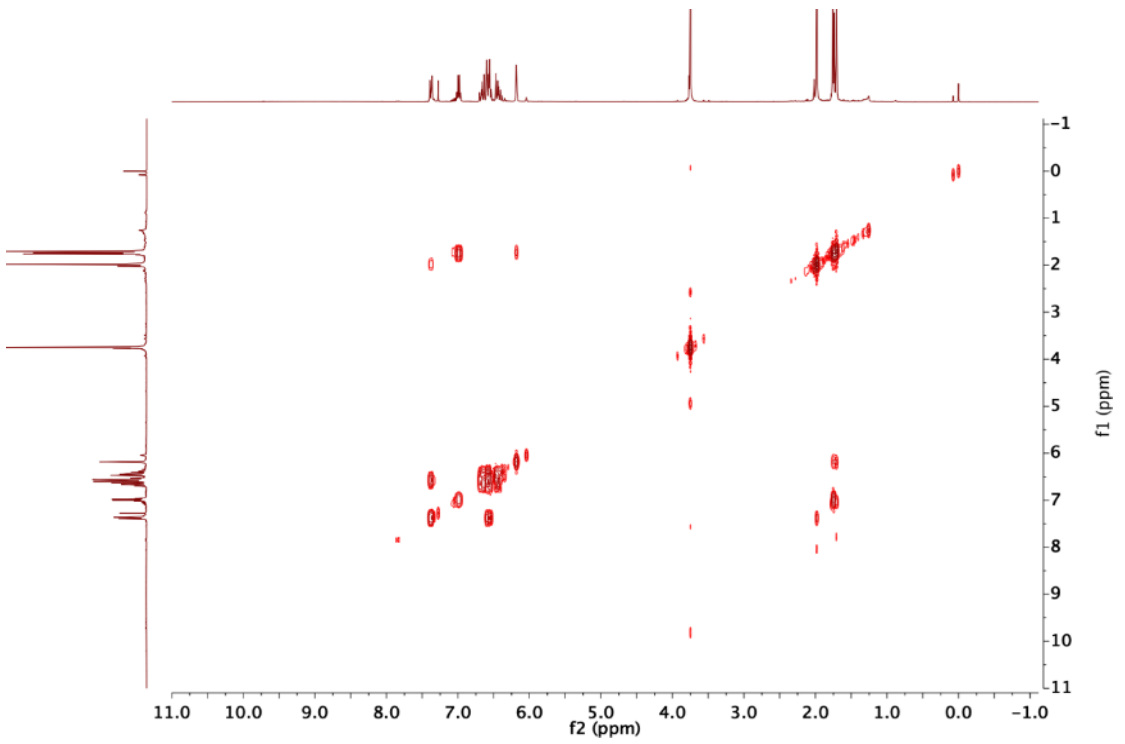

Figure S 25. ^{1}H-^{1}H COSY spectrum of 3 in $CDCl_3$.

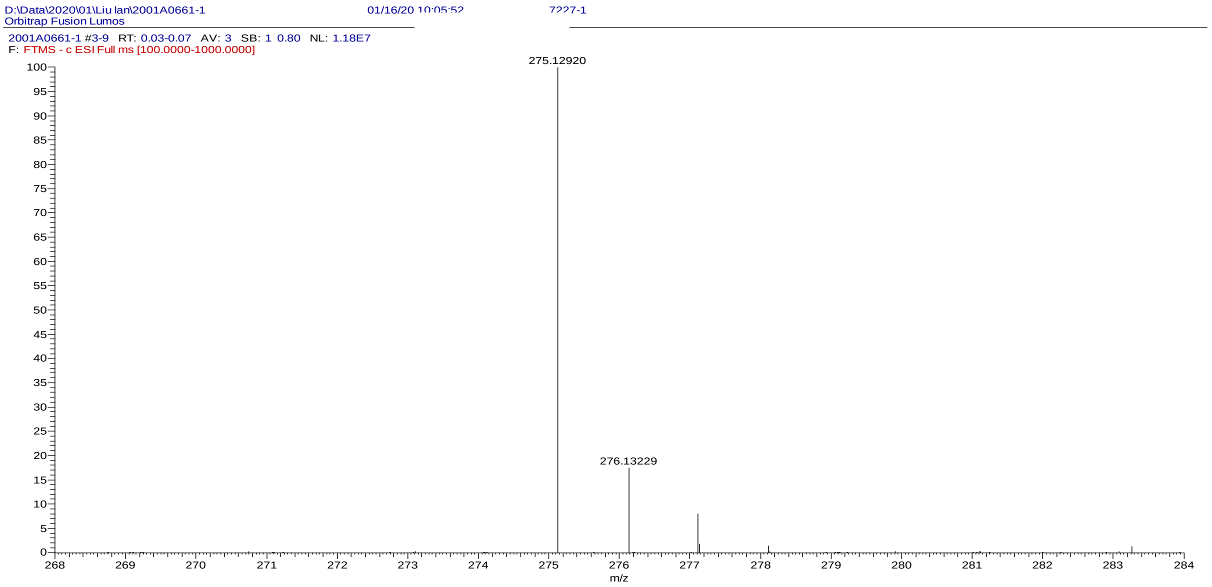

SPECTRUM - simulation :

m/z	Theo. Mass	Delta (ppm)	RDB equiv.	Composition
275.12920	275.12888	1.15	7.5	C16 H19 O4

Figure S 26. HR-ESIMS spectrum of 3.

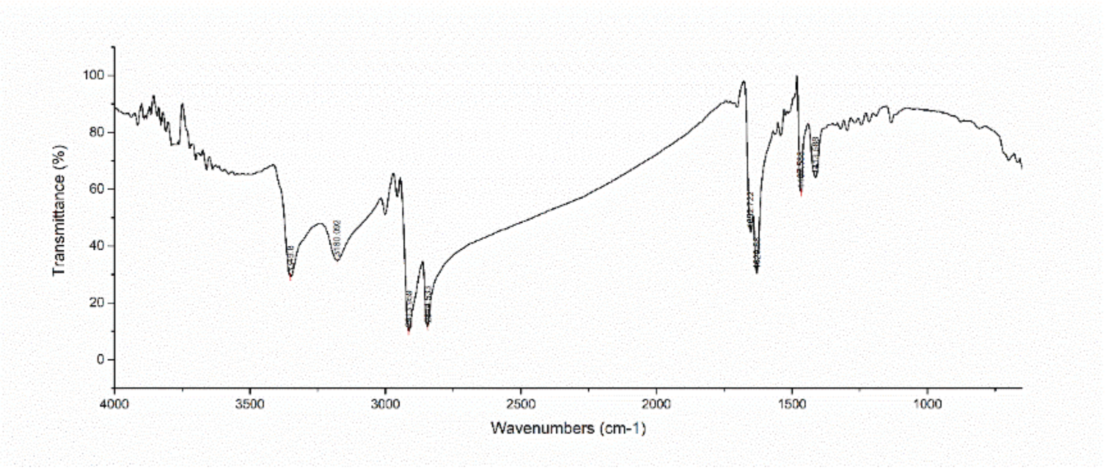

Figure S 27. IR spectrum of 3.

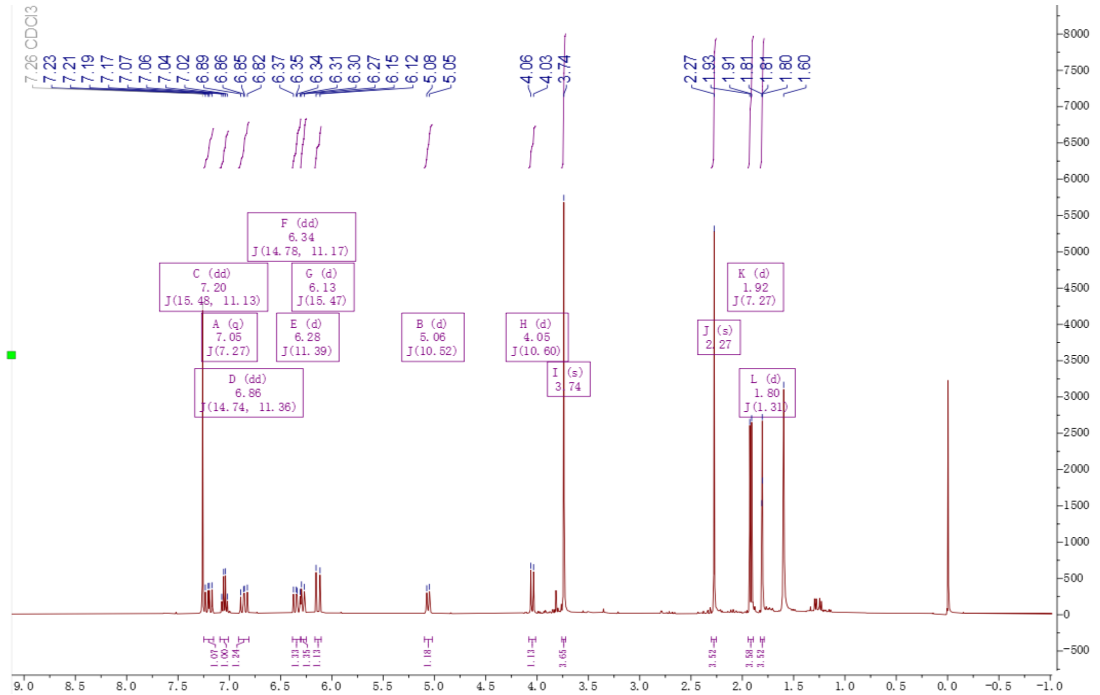

Figure S 28. ^{1}H NMR spectrum of 4 in $CDCl_3$.

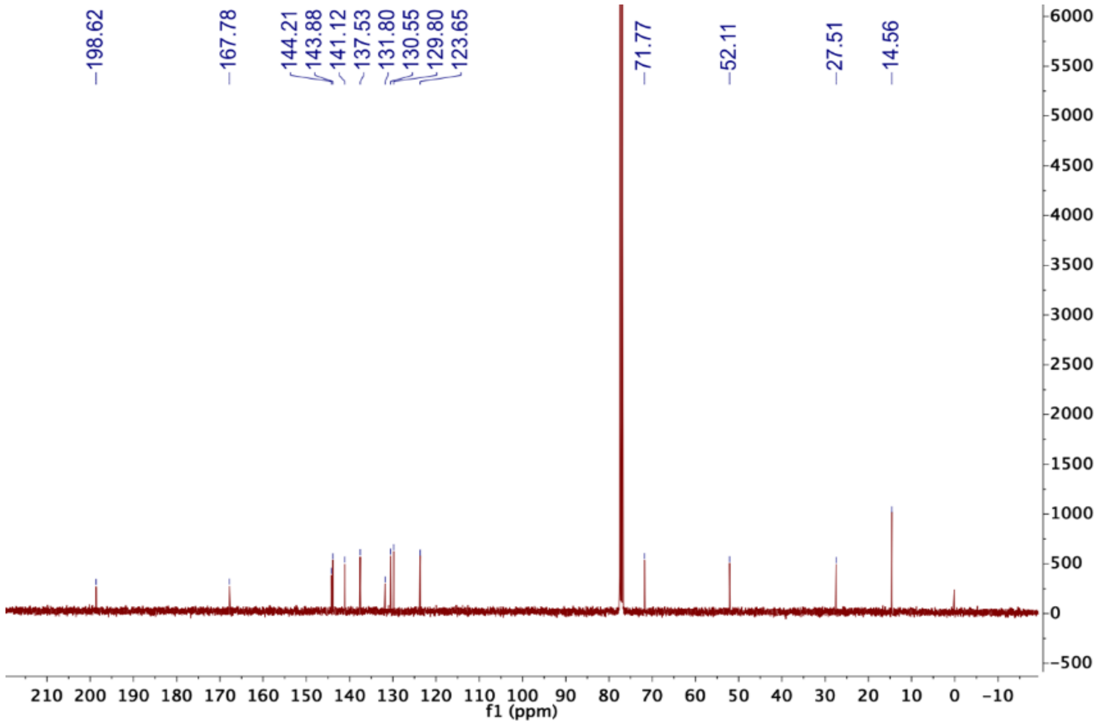

Figure S 29. ^{13}C NMR spectrum of 4 in $CDCl_3$.

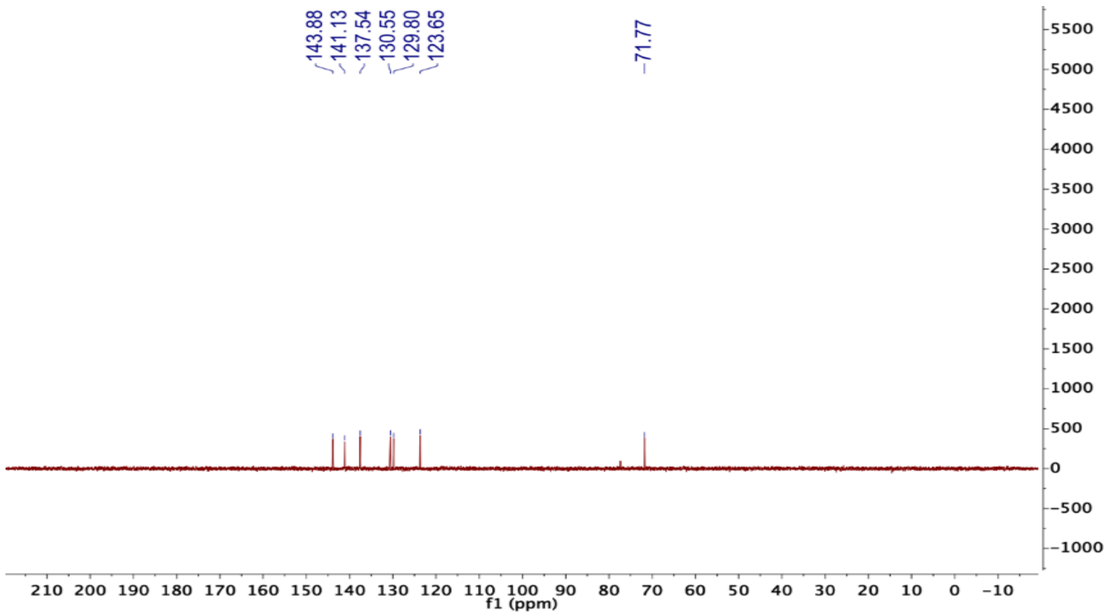

Figure S 30. DEPT-90 spectrum of 4 in $CDCl_3$.

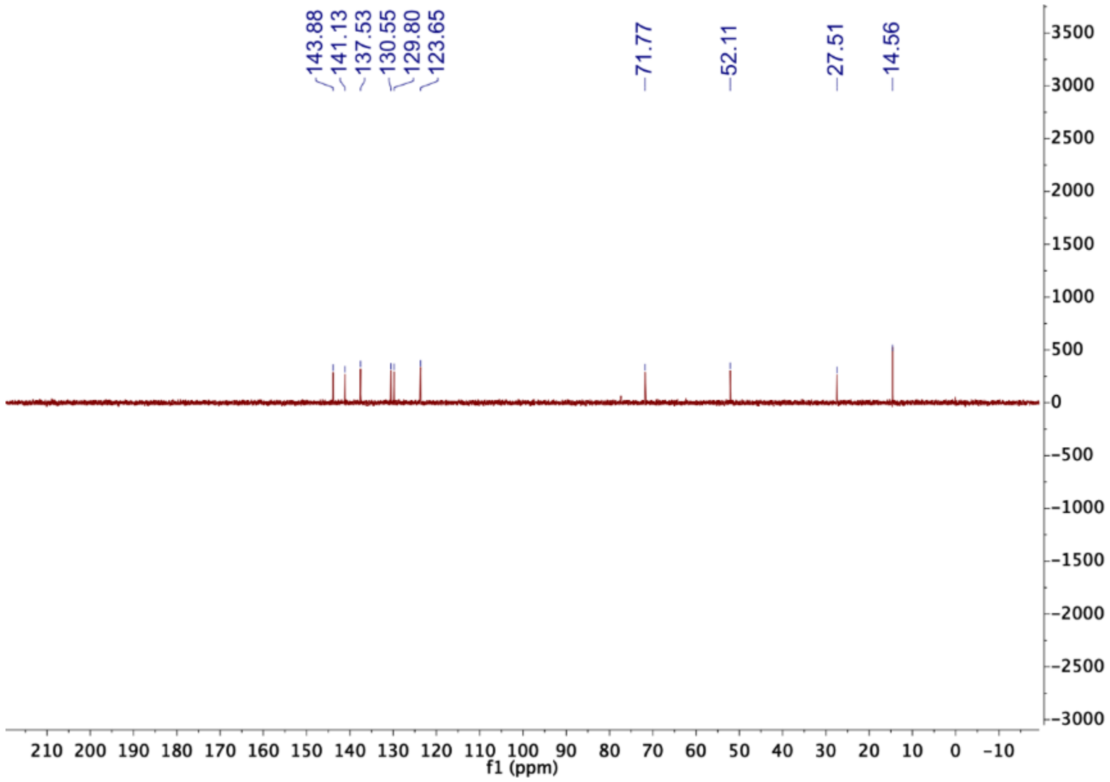

Figure S 31. DEPT-135 spectrum of 4 in $CDCl_3$.

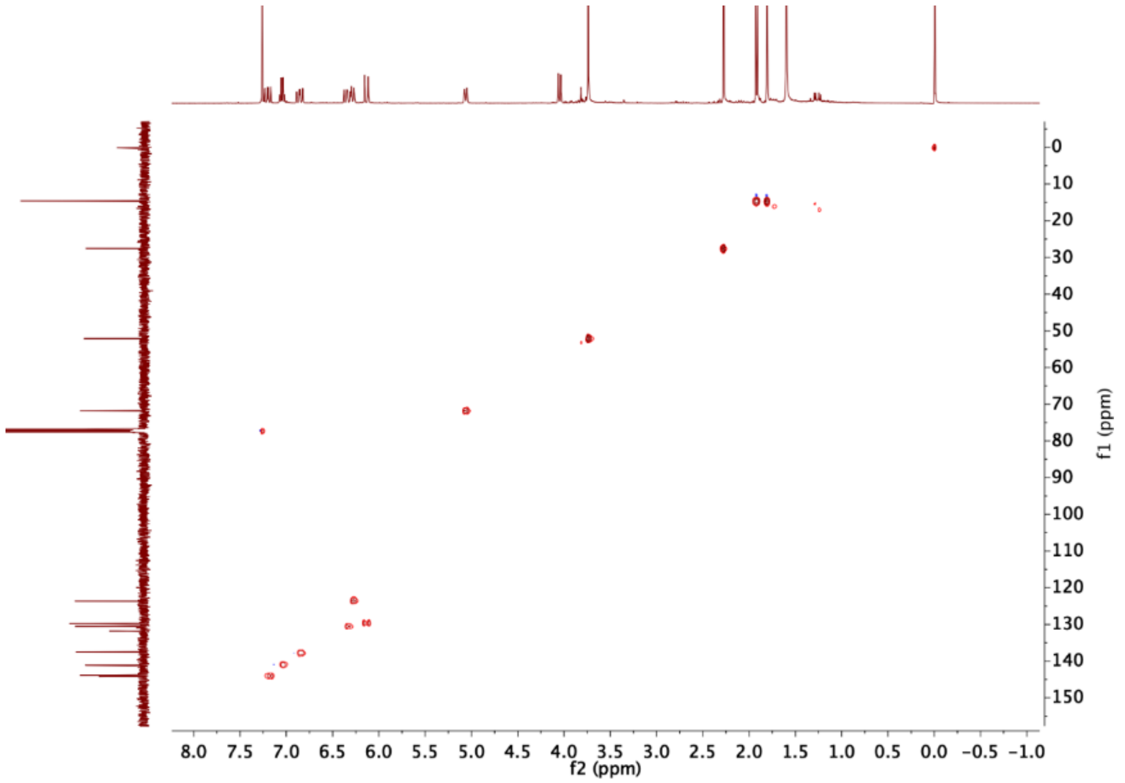

Figure S 32. HSQC spectrum of 4 in $CDCl_3$.

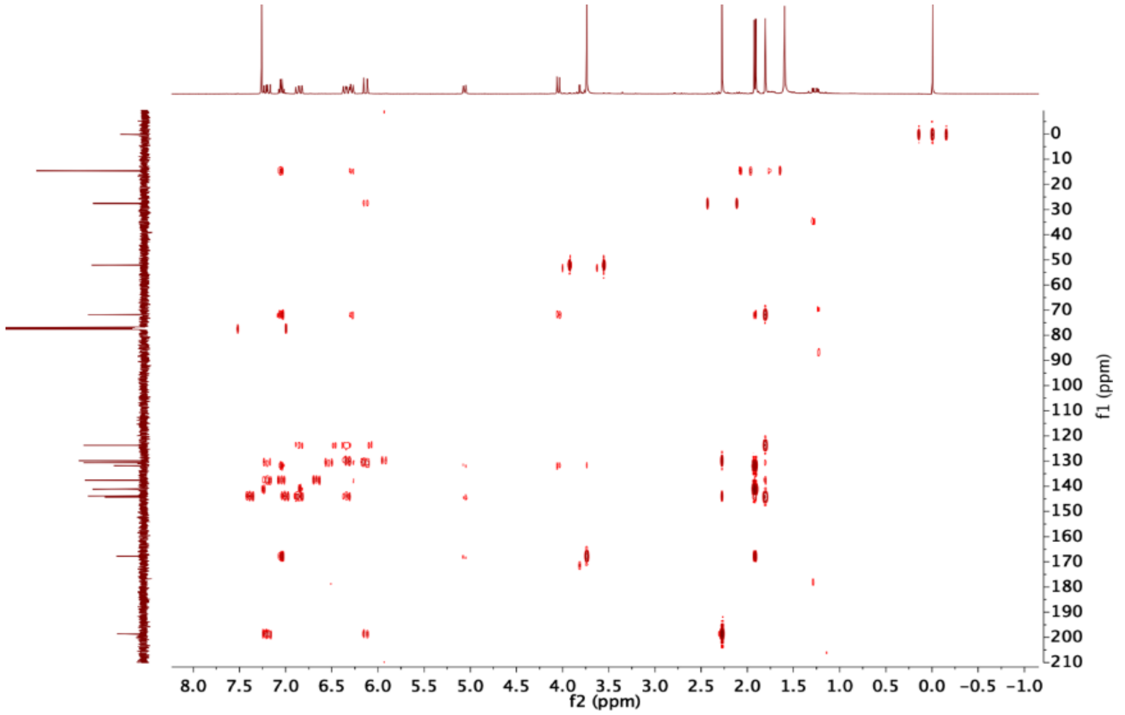

Figure S 33. HMBC spectrum of 4 in $CDCl_3$.

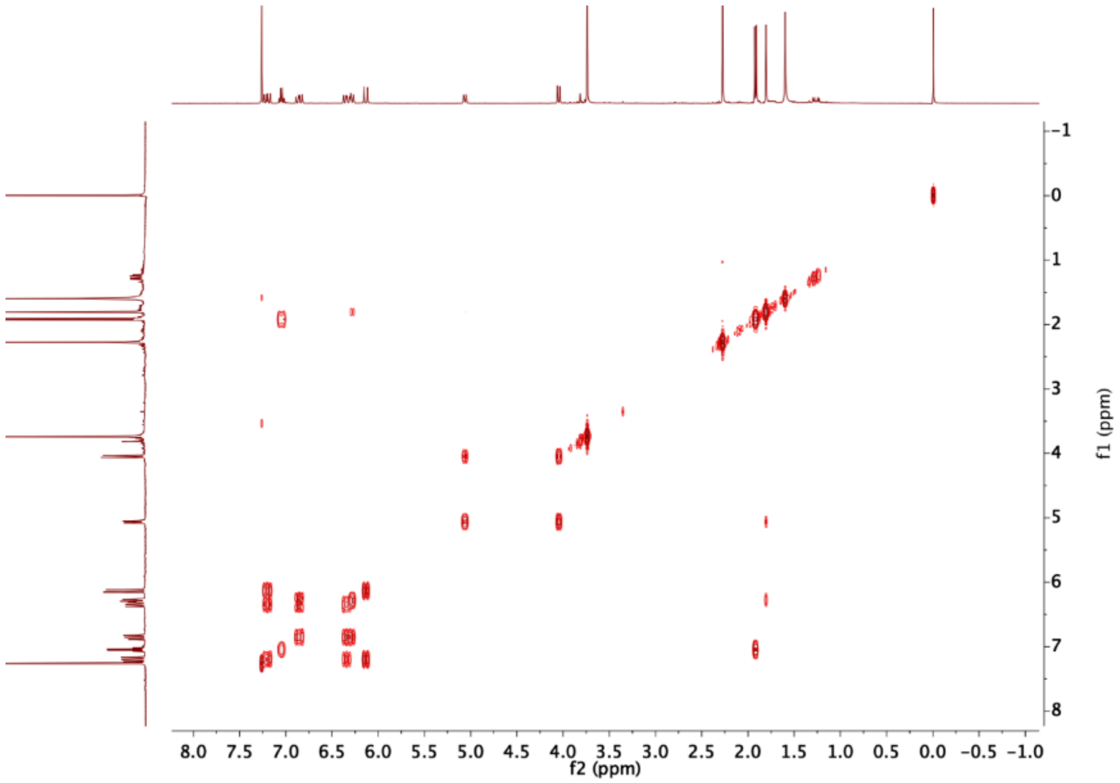

Figure S 34. **^{1}H-^{1}H COSY spectrum of 4 in $CDCl_3$.**

Analysis Info

Analysis Name	D:\Data\2020\05\Liu lan\2005A0279\2005A0279_7227-4_pos_RA4_01_3547.d	Acquisition Date	5/22/2020 12:27:35 PM	
Method	Tune_pos_low_LCMS.m	Operator	Demo User	
Sample Name	2005A0279_7227-4_pos	Instrument	timsTOF	1844426.00062
Comment				

Acquisition Parameter

Source Type	ESI	Ion Polarity	Positive	Set Nebulizer	0.6 Bar
Focus	Not active	Set Capillary	4500 V	Set Dry Heater	200 °C
Scan Begin	100 m/z	Set End Plate Offset	-500 V	Set Dry Gas	6.0 l/min
Scan End	1000 m/z	Set Collision Cell RF	500.0 Vpp	Set Divert Valve	Waste

+MS, 0.10-0.17min

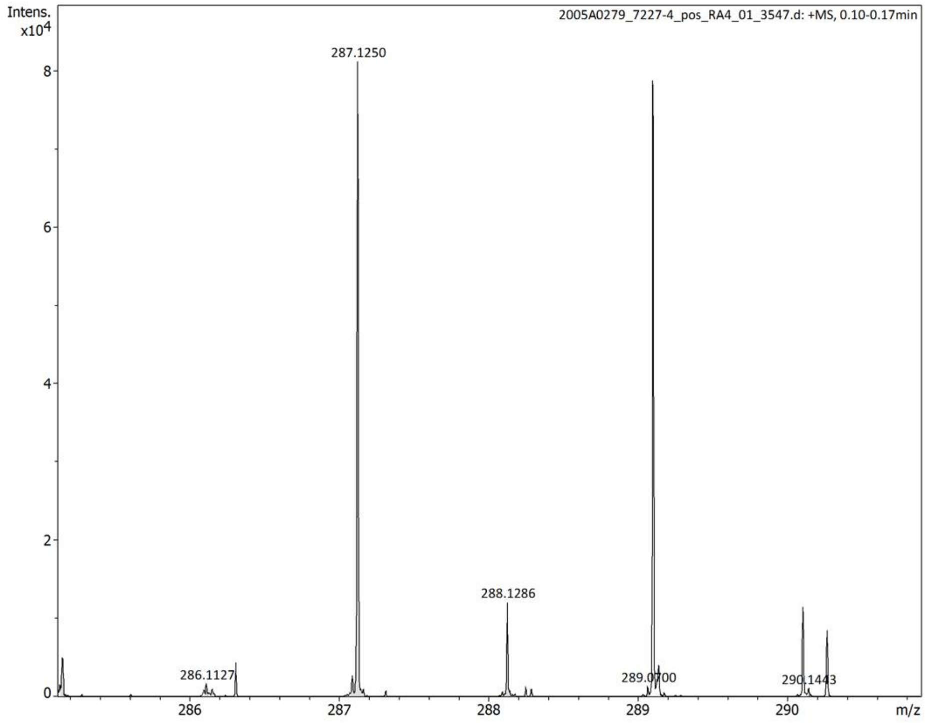

Meas. m/z	Ion Formula	m/z	z	err [ppm]	err [mDa]	mSigma	rdb	e⁻ Conf	N-Rule
287.1250	C15H20NaO4	287.1254	1+	1.2	0.3	19.3	6.0	even	ok

Figure S 35. HR-ESIMS spectrum of 4.

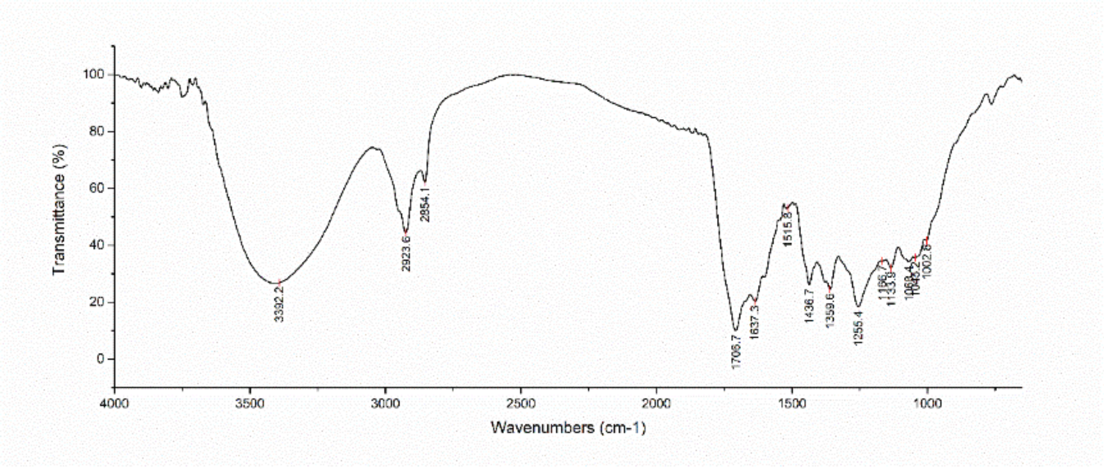

Figure S 36. IR spectrum of 4.

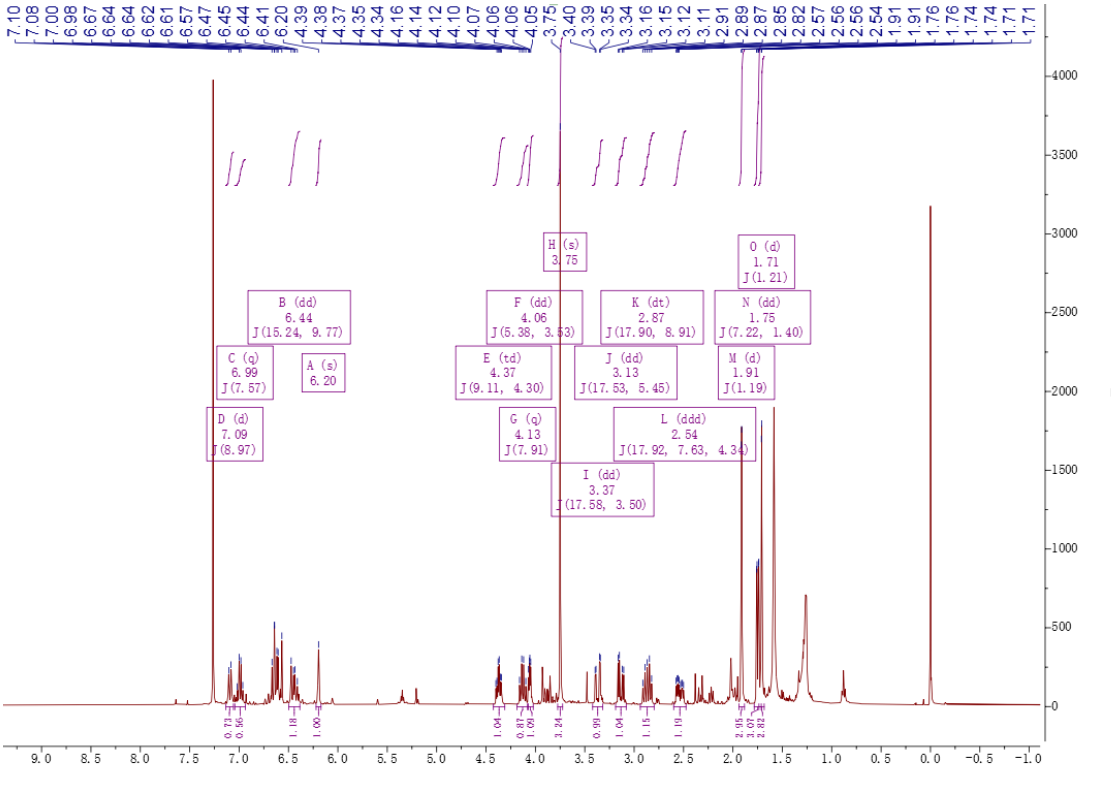

Figure S 37. ^{1}H NMR spectrum of 5 in $CDCl_3$.

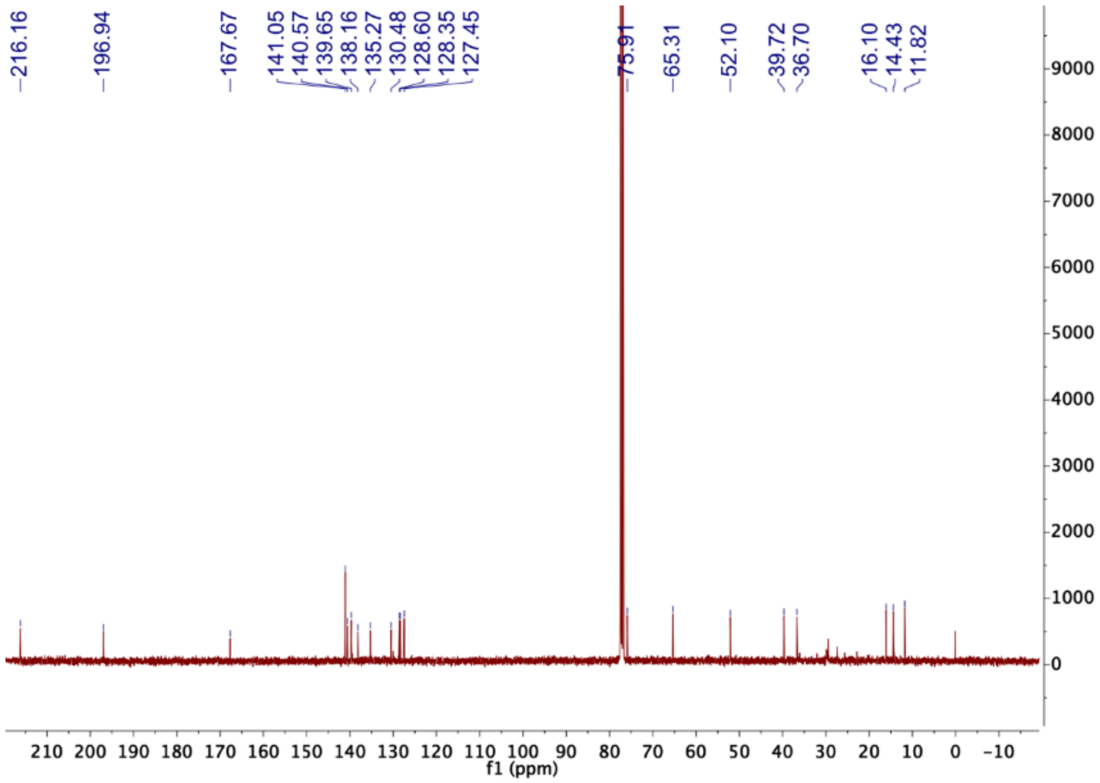

Figure S 38. ^{13}**C NMR spectrum of 5 in CDCl**$_3$**.**

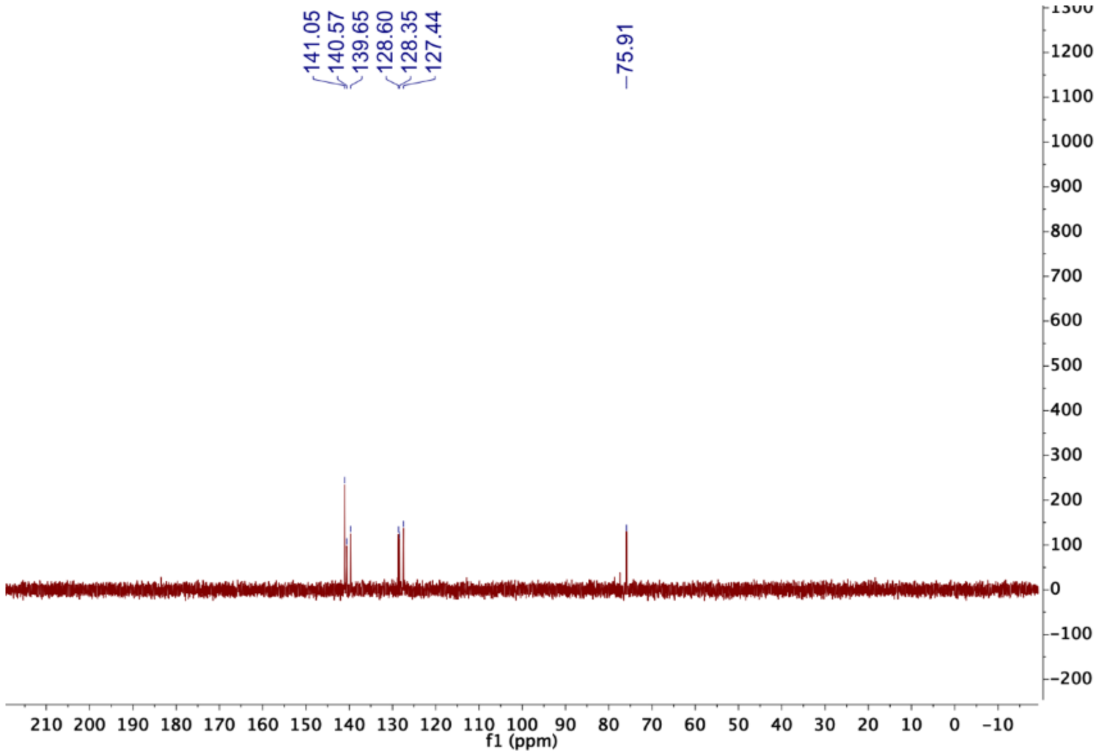

Figure S 39. DEPT-90 spectrum of 5 in CDCl$_3$**.**

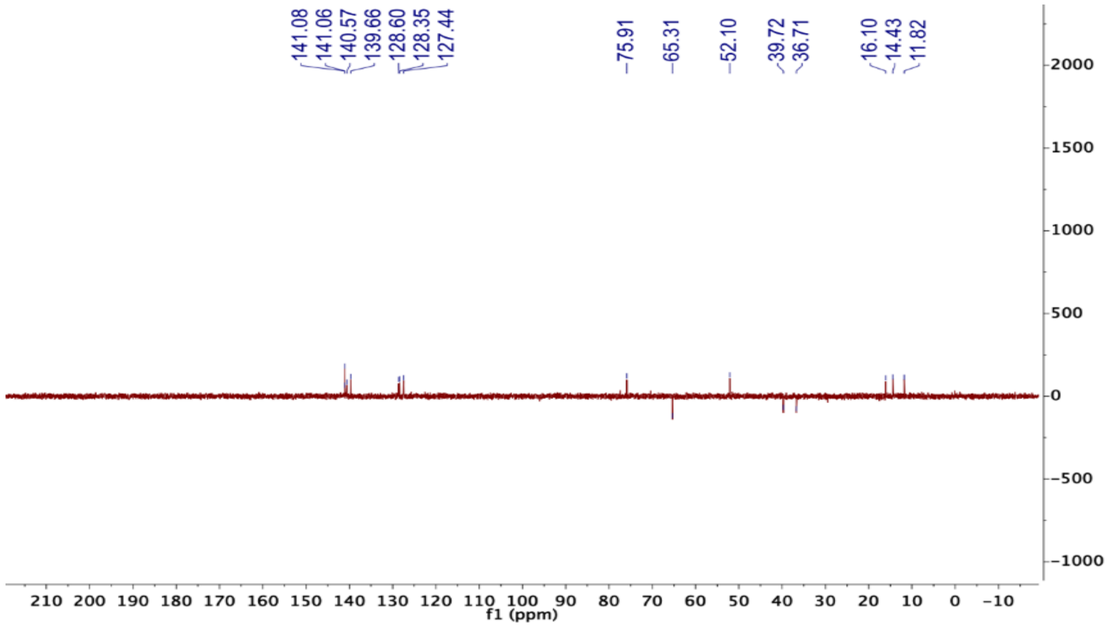

Figure S 40. DEPT-135 spectrum of 5 in CDCl$_3$.

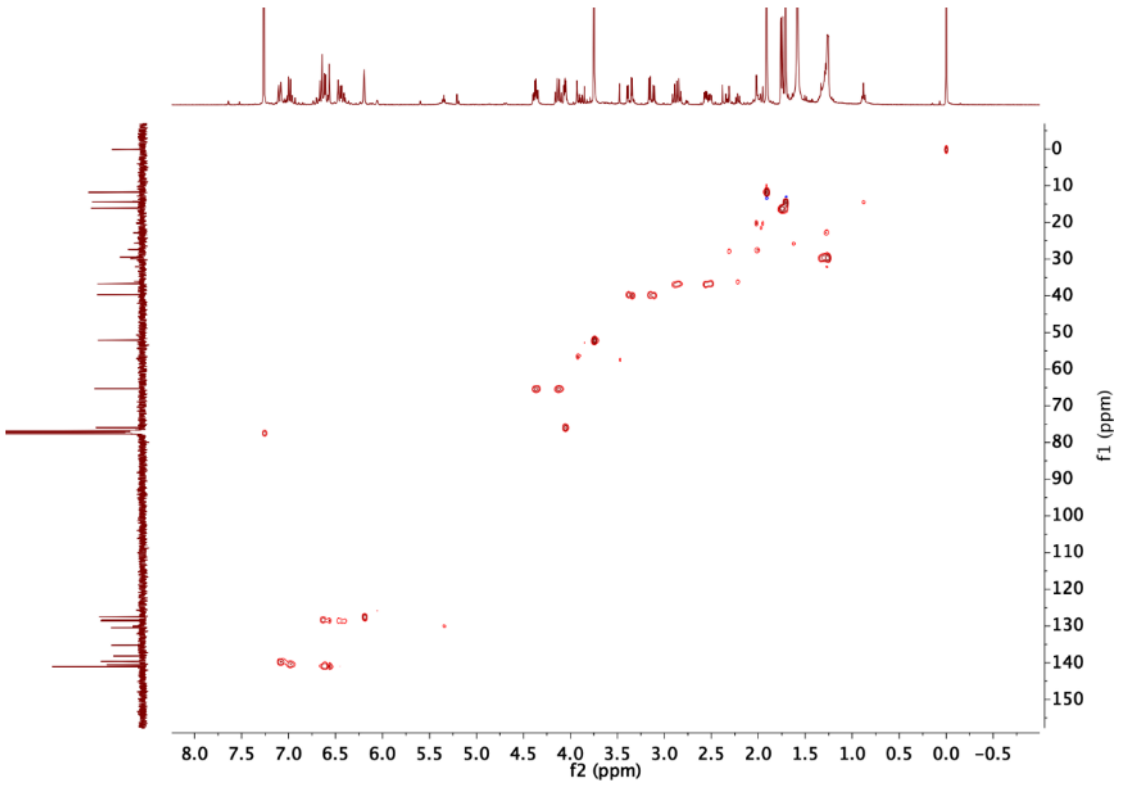

Figure S 41. HSQC spectrum of 5 in CDCl$_3$.

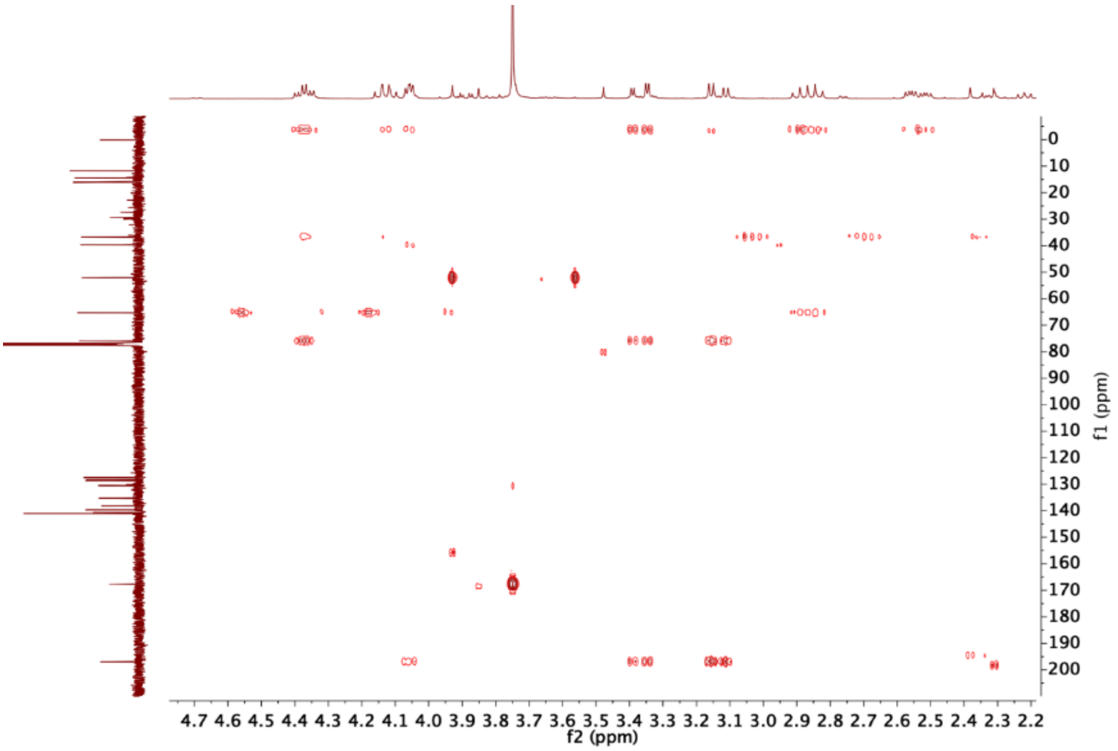

Figure S 42. HMBC spectrum of 5 in $CDCl_3$.

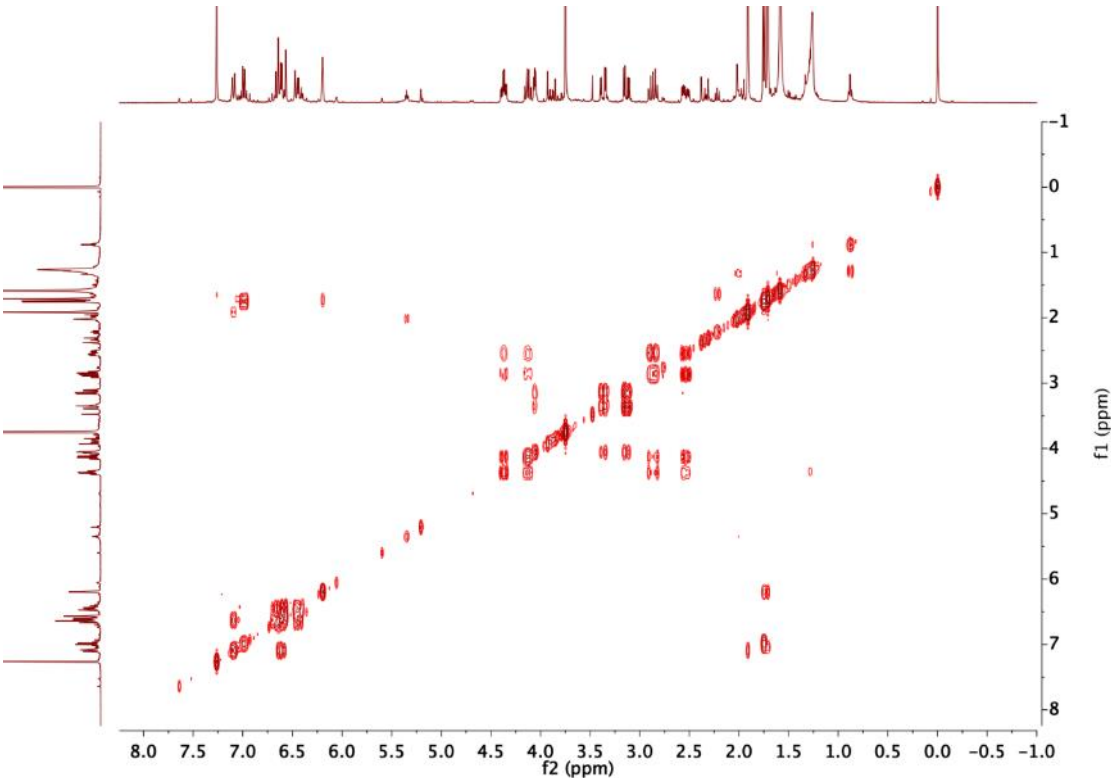

Figure S 43. ^{1}H-^{1}H COSY spectrum of 5 in $CDCl_3$.

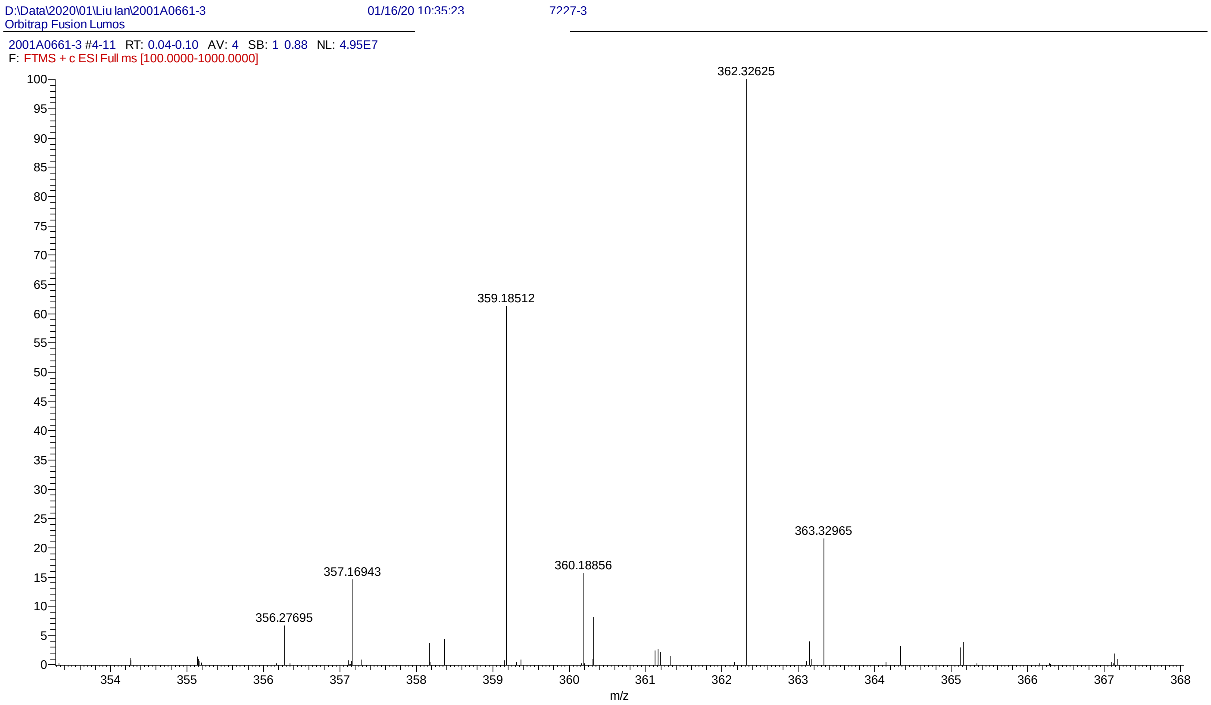

SPECTRUM - simulation :

m/z	Theo. Mass	Delta (ppm)	RDB equiv.	Composition
359.18512	359.1853	-0.50	8.5	C21 H27 O5

Figure S 44. HR-ESIMS spectrum of **5**.

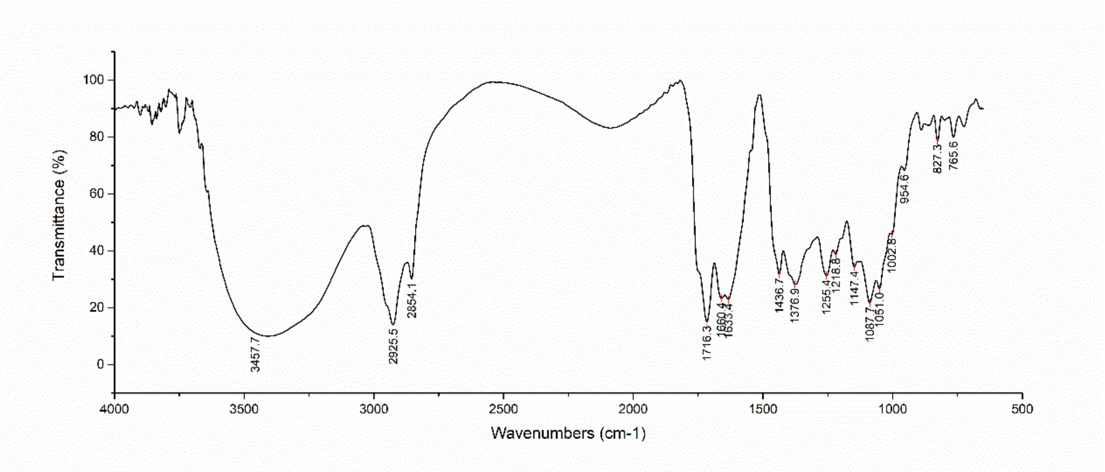

Figure S 45. IR spectrum of 5.

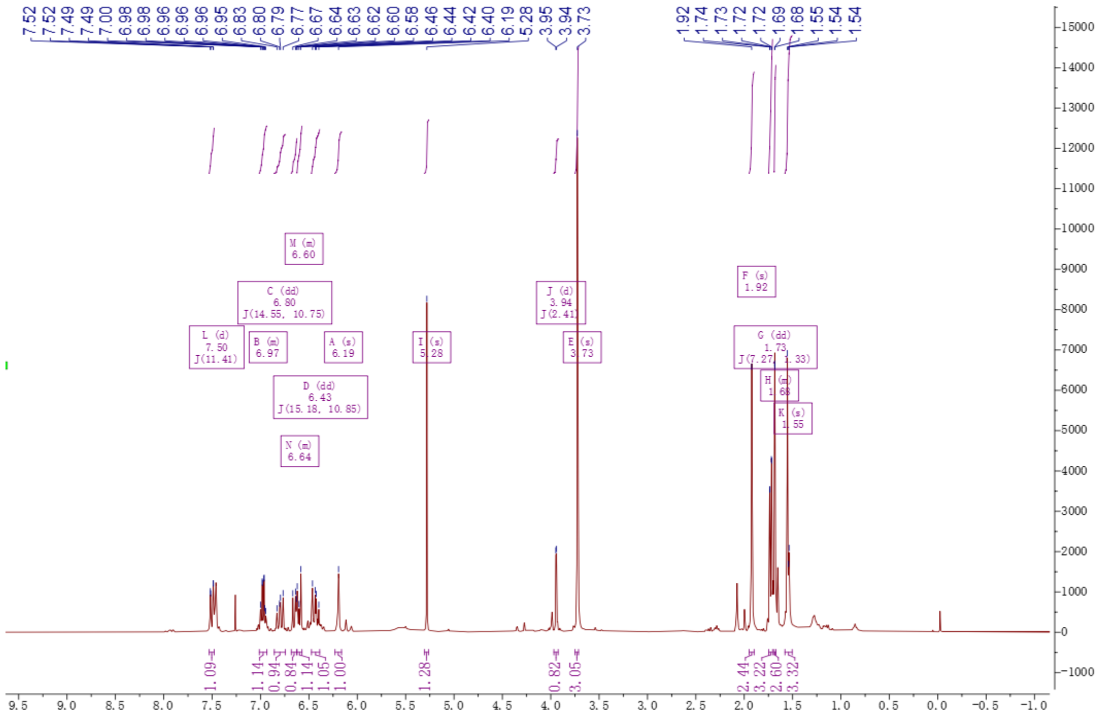

Figure S 46. ^{1}H NMR spectrum of 6 in $CDCl_3$.

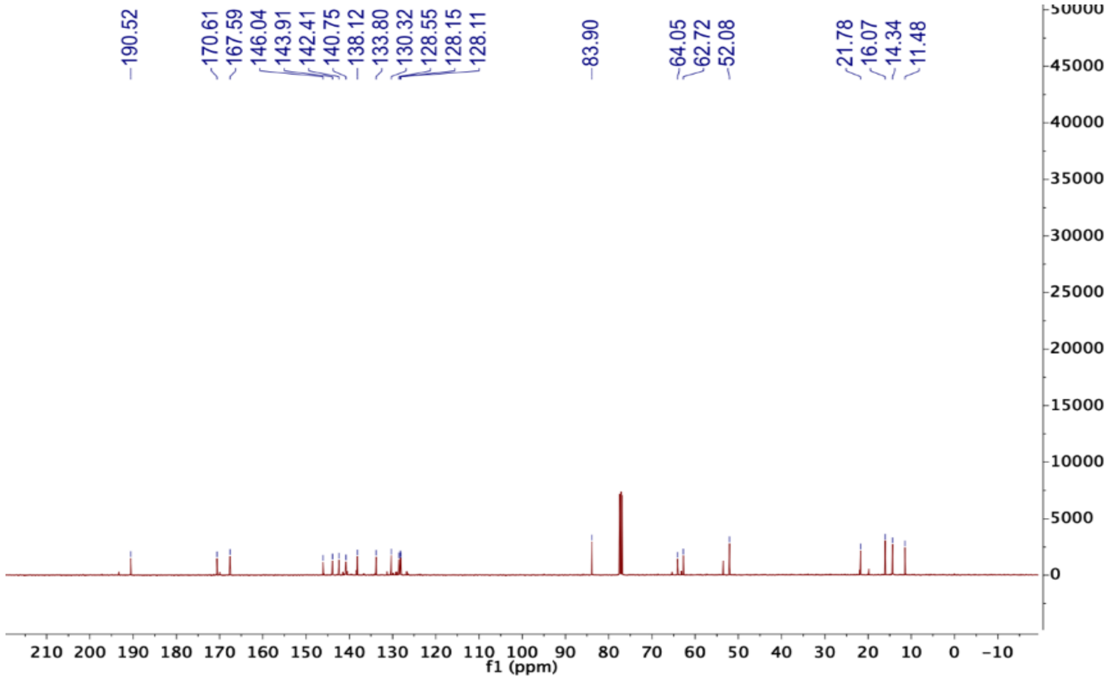

Figure S 47. ^{13}C NMR spectrum of 6 in $CDCl_3$.

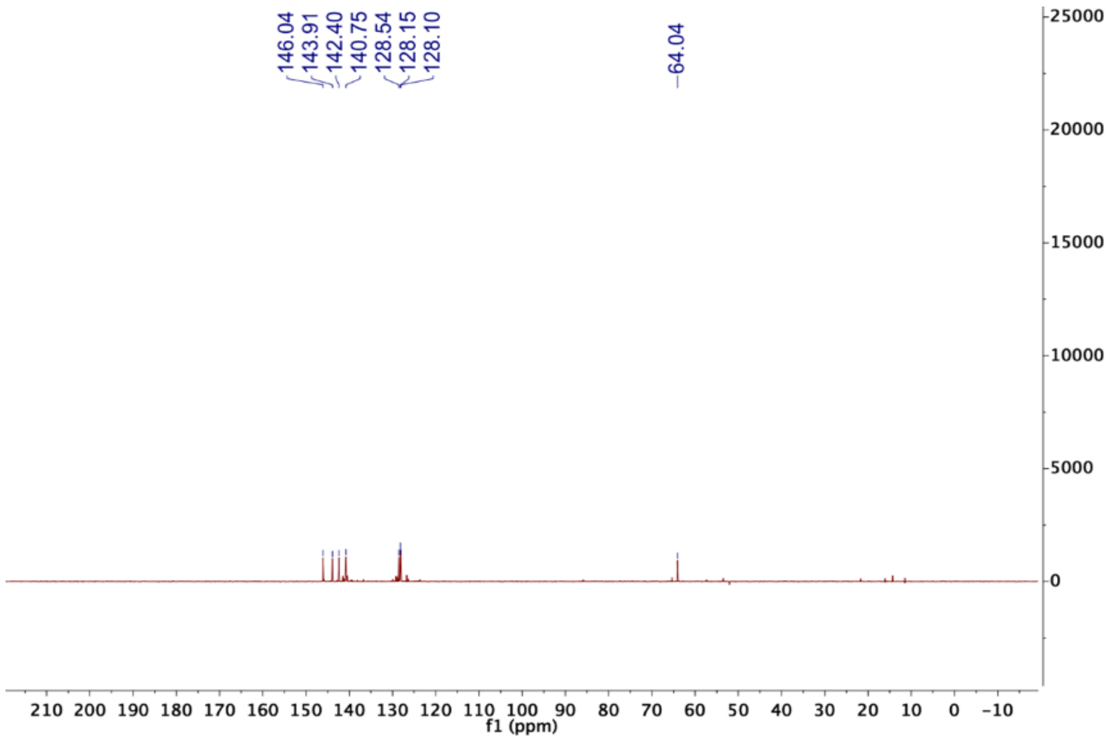

Figure S 48. DEPT-90 spectrum of 6 in $CDCl_3$.

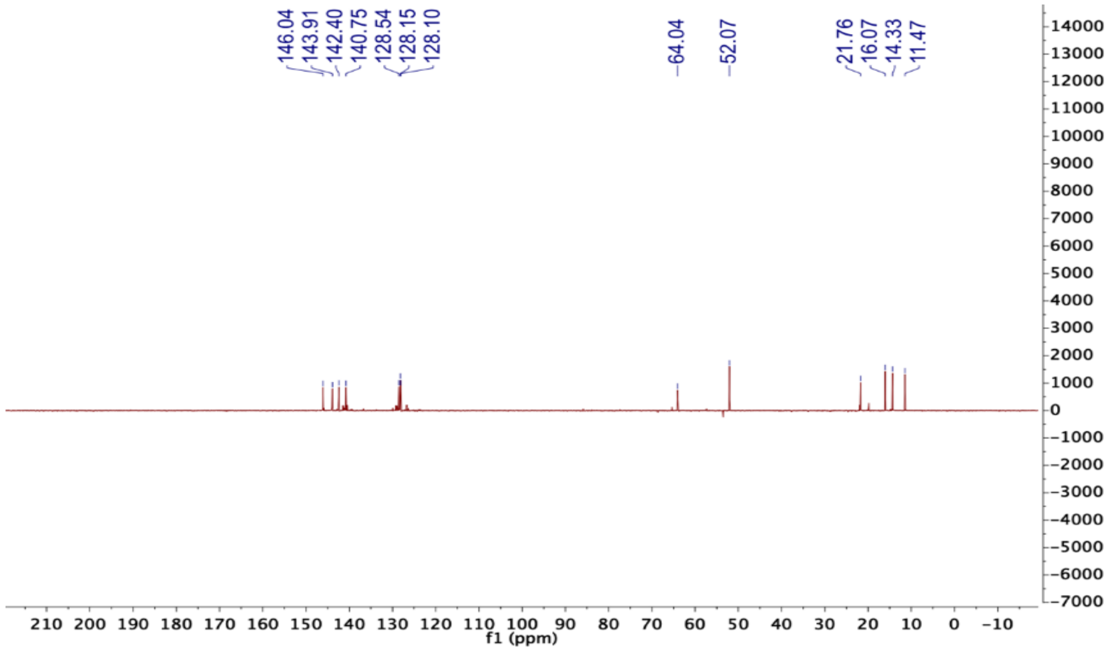

Figure S 49. DEPT-135 spectrum of 6 in $CDCl_3$.

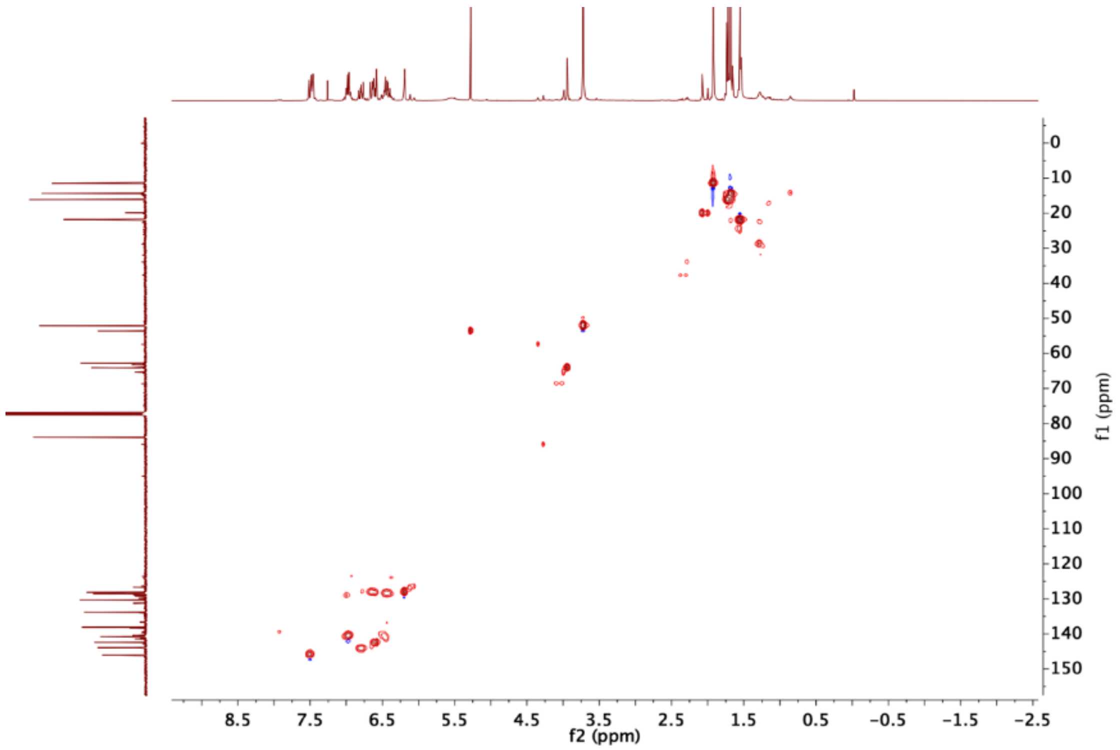

Figure S 50. HSQC spectrum of 6 in $CDCl_3$.

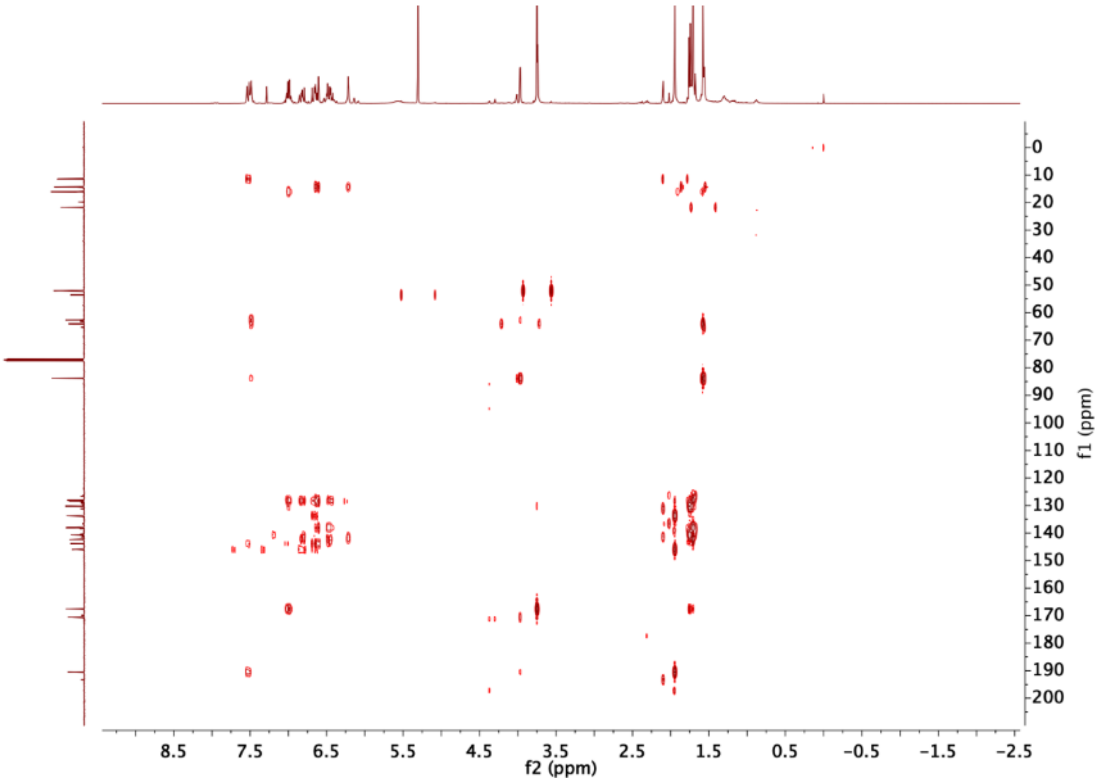

Figure S 51. HMBC spectrum of 6 in $CDCl_3$.

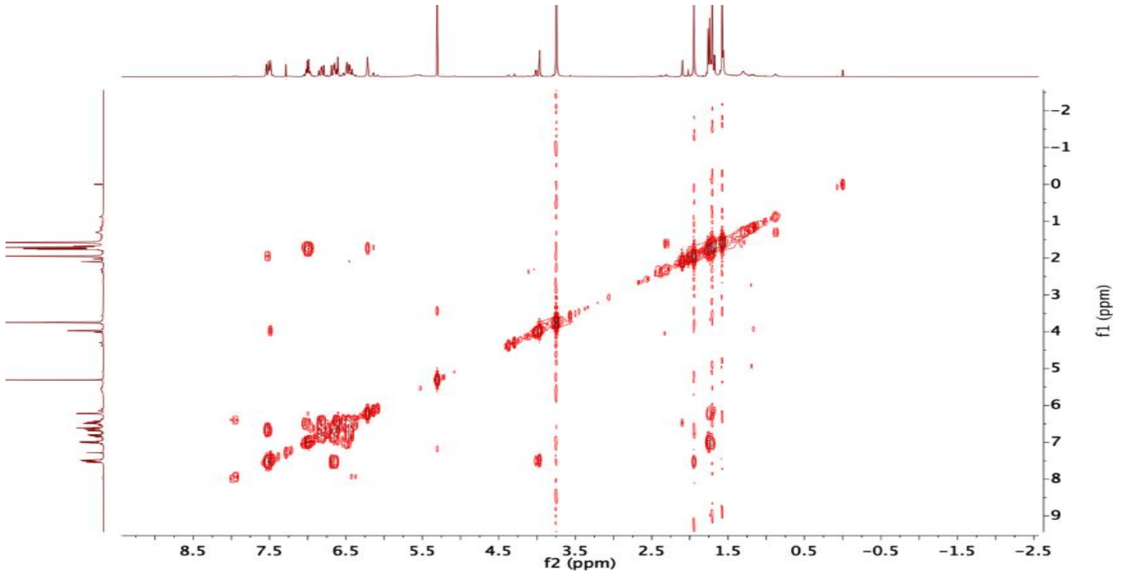

Figure S 52. ^{1}H-^{1}H COSY spectrum of 6 in $CDCl_3$.

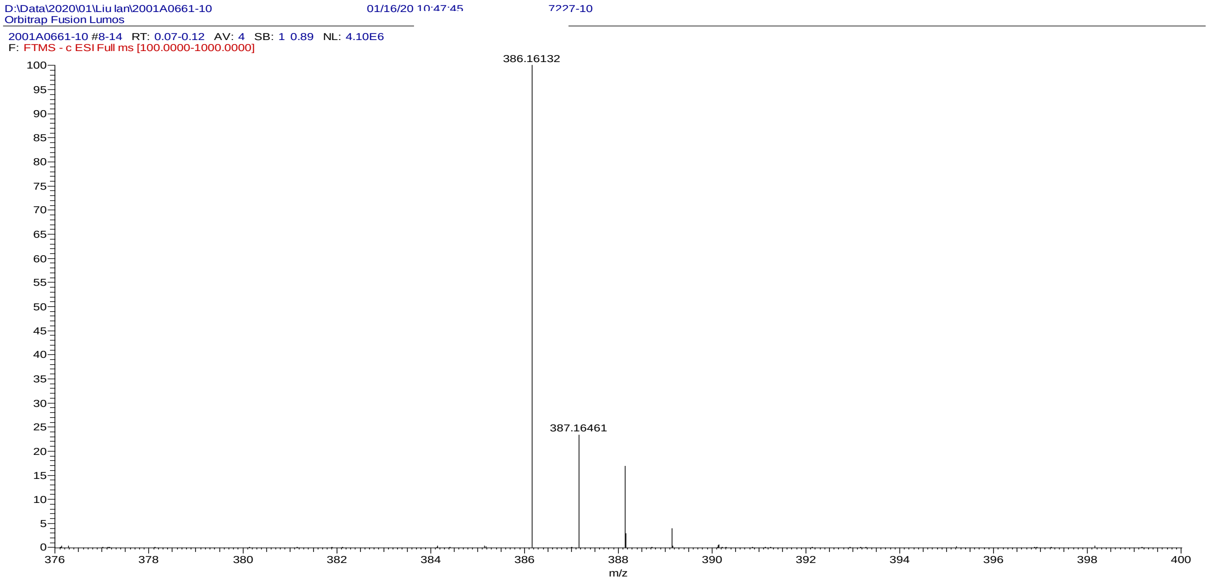

SPECTRUM - simulation :

m/z	Theo. Mass	Delta (ppm)	RDB equiv.	Composition
386.16132	386.16091	1.06	10.5	C21 H24 O6 N

Figure S 53. HR-ESIMS spectrum of 6.

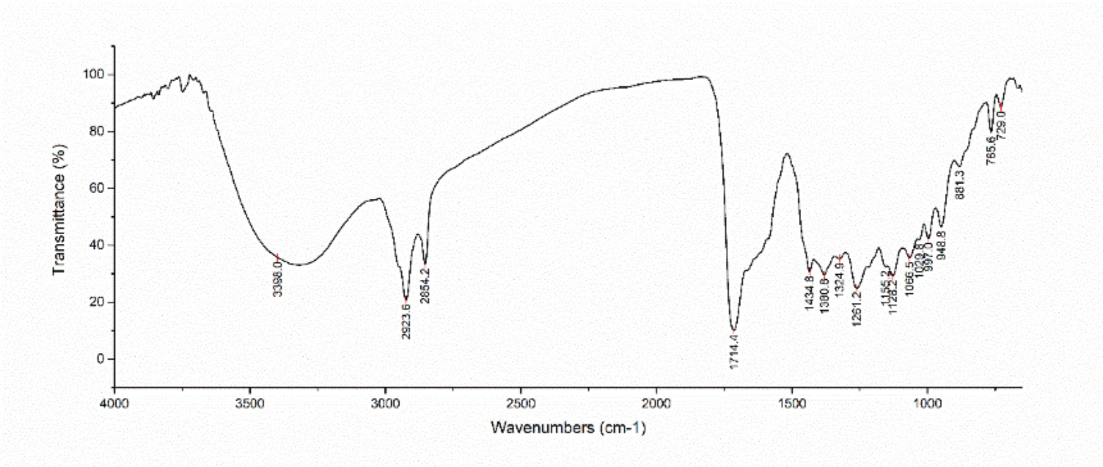

Figure S 54. IR spectrum of 6.

Table S1. Energy Analysis for the Conformers of (5*S*)-2.

Compund	E(Hartree)	E(kcal/mol)	rel.E(kcal/mol)	Qi	Boltzmann dist
2-a	-652.338	-409348	0	1	0.750132
2-b	-652.335	-409346	2.170371	0.025589	0.019195
2-c	-652.334	-409345	2.87516	0.007782	0.005838
2-d	-652.337	-409348	0.713406	0.299727	0.224835

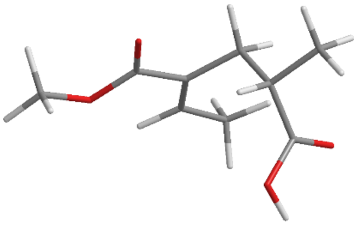

2-a

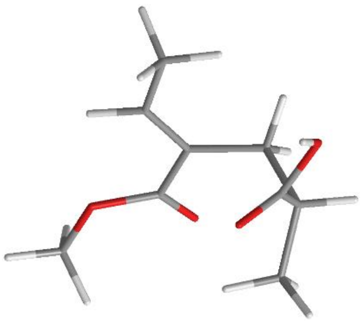

2-b

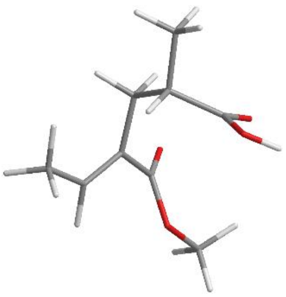

2-c

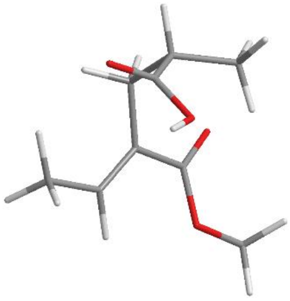

2-d

Figure S 55. The optimized low energy conformers of 2.

Table S2. Energy Analysis for the Conformers of (4*S*)-4.

	E(Hartree)	E(kcal/mol)	rel.E(kcal/mol)	Qi	Boltzmann dist
4-a	-884.534	-555053	0	1	0.072528
4-b	-884.534	-555053	-0.13286	1.251554	0.090773
4-c	-884.533	-555053	0.339281	0.563822	0.040893
4-d	-884.533	-555053	0.193439	0.721298	0.052314
4-e	-884.534	-555054	-0.40674	1.987644	0.14416
4-f	-884.534	-555054	-0.52816	2.440049	0.176972
4-g	-884.535	-555054	-0.57549	2.643116	0.1917
4-h	-884.535	-555054	-0.68504	3.180275	0.230659

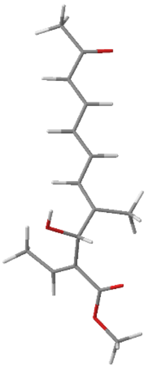

4-a

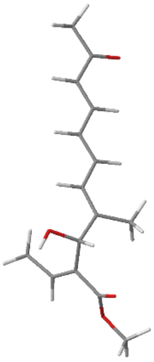

4-b

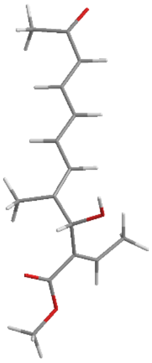

4-c

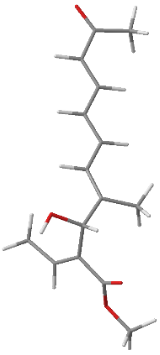

4-d

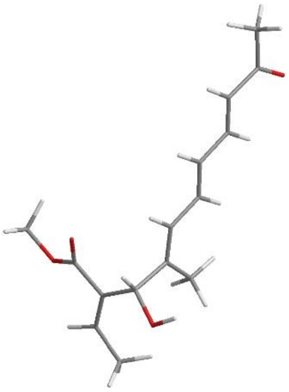

4-e

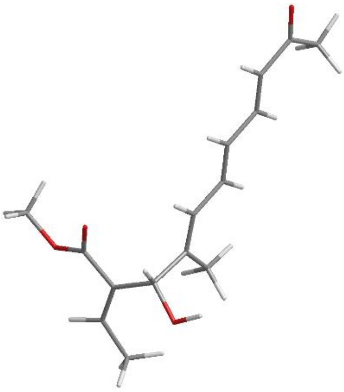

4-f

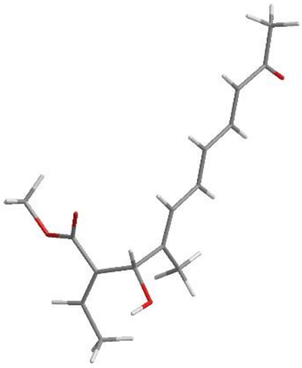

4-g

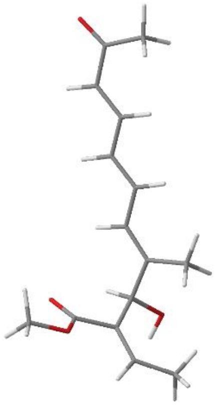

4-h

Figure S 56. The optimized low energy conformers of 4.

Table S3. Energy Analysis for the Conformers of (13*S*)-5.

	E(Hartree)	E(kcal/mol)	rel.E(kcal/mol)	Qi	Boltzmann dist
5-a	-1192	-747992	0	1	0.066811
5-b	-1192	-747992	0.43718	0.477897	0.031929
5-c	-1192	-747991	1.224158	0.126502	0.008452
5-d	-1192	-747991	1.62048	0.064774	0.004328
5-e	-1192	-747992	0.256187	0.648769	0.043345
5-f	-1192	-747992	0.271373	0.632342	0.042247
5-g	-1192	-747993	-1.05911	5.981959	0.399658
5-h	-1192	-747993	-1.03904	5.782611	0.386339
5-i	-1192	-747991	0.814131	0.25284	0.016892

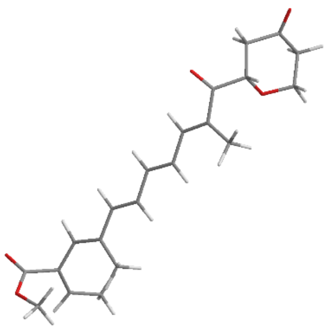

5-a

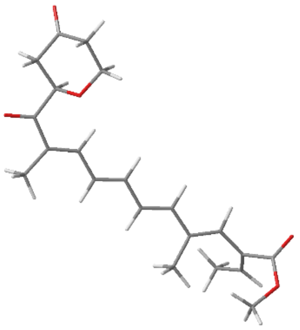

5-b

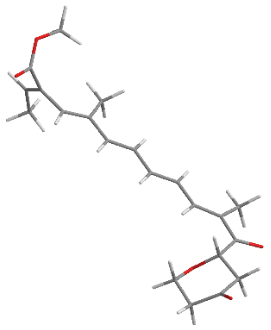

5-c

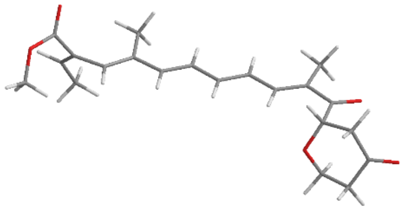

5-d

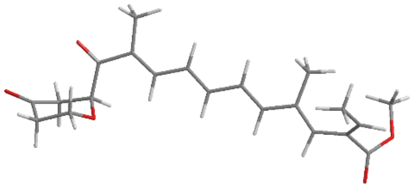

5-e

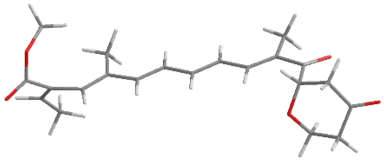

5-f

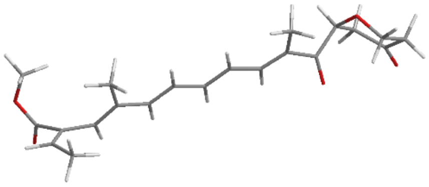

5-g

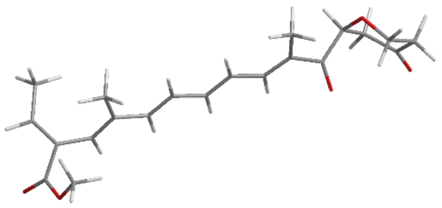

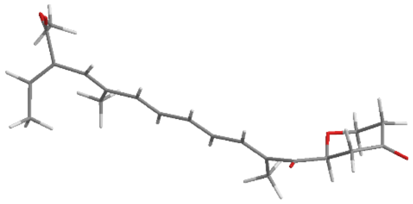

5-h **5-i**

Figure S 57. The optimized low energy conformers of 5.

Table S4. Energy Analysis for the Conformers of (13*R*, 14*R*,15*S*)-6.

Compund	E(Hartree)	E(kcal/mol)	rel.E(kcal/mol)	Qi	Boltzmann dist
6-a	-1321.34	-829153	0	1	0.781632
6-b	-1321.34	-829152	1.327032	0.106326	0.083108
6-c	-1321.33	-829148	4.882758	0.000262	0.000205
6-d	-1321.33	-829146	6.788994	1.05E-05	8.19E-06
6-e	-1321.33	-829148	5.636861	7.34E-05	5.73E-05
6-f	-1321.34	-829150	2.943804	0.00693	0.005417
6-g	-1321.33	-829147	5.748432	6.08E-05	4.75E-05
6-h	-1321.34	-829152	1.0643	0.165711	0.129525

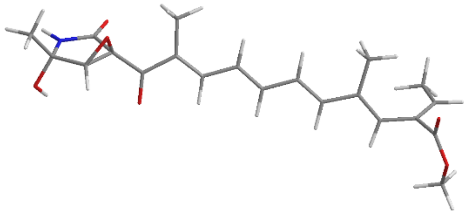

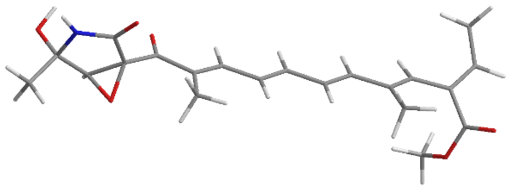

6-a **6-b**

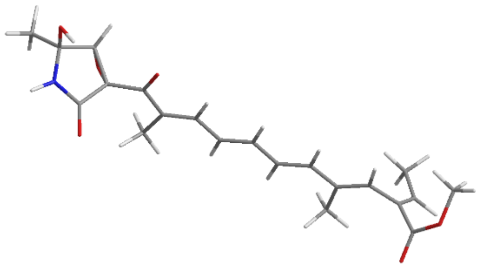

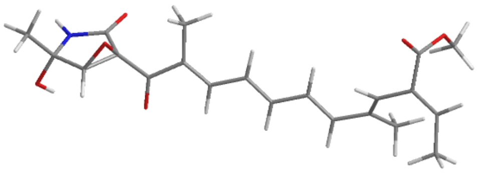

6-c **6-d**

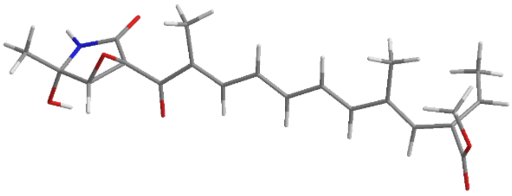

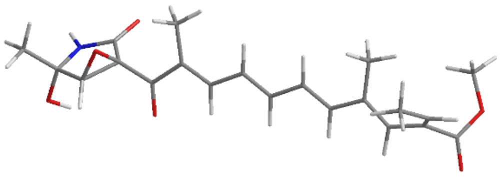

6-e 6-f

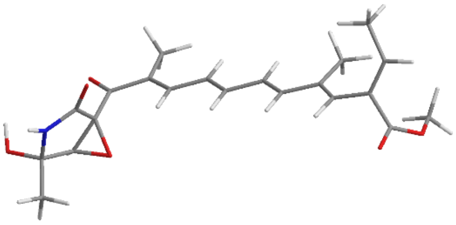

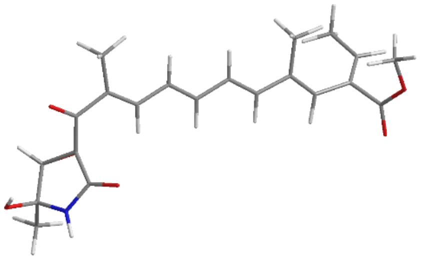

6-g 6-h

Figure S 58. The optimized low energy conformers of (13*R*, 14*R*,15*S*)-6.

Table S5. Energy Analysis for the Conformers of (13*R*, 14*R*,15*R*)-6.

Compund	E(Hartree)	E(kcal/mol)	rel.E(kcal/mol)	Qi	Boltzmann dist
6-i	− 1320. 7589508500	− 828788. 7479	0	1	0. 015356
6-j	− 1320. 7587168500	− 828788. 6011	0. 146837216	0. 780363	0. 011983
6-k	− 1320. 7618523300	− 828790. 5686	−1. 820706174	21. 65029	0. 33246
6-l	− 1320. 7618578700	− 828790. 5721	−1. 824182577	21. 77778	0. 334417
6-m	− 1320. 7617734100	− 828790. 5191	−1. 771183127	19. 91312	0. 305784

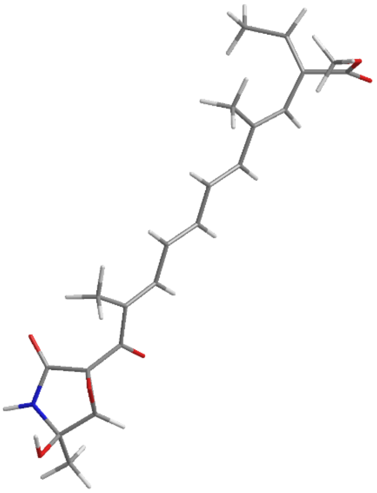

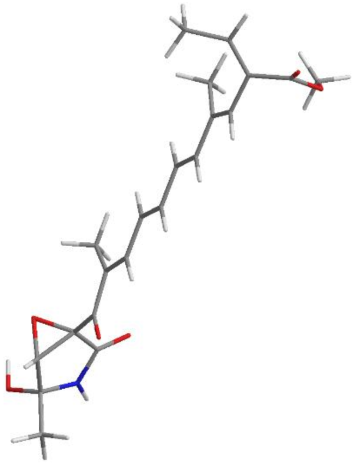

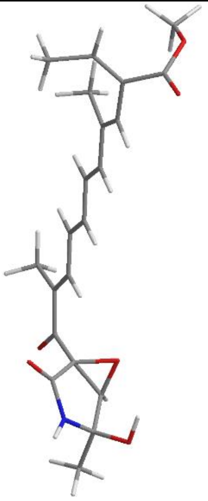

6-i **6-j** **6-k**

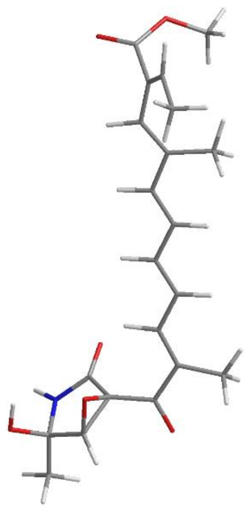

6-l

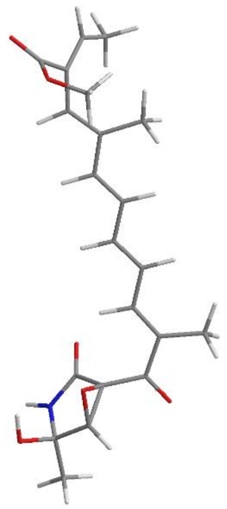

6-m

Figure S 59. The optimized low energy conformers of (13*R*, 14*R*,15*R*)-6.
